# BioFRAM PGx, an Implementation Proposal for Pharmacogenetics in the Spanish National Health System

**DOI:** 10.3390/ph19081305

**Published:** 2026-08-18

**Authors:** Francisco Arias-Aragón, Carmen Mata-Martín, Alba Sánchez-Redondo, Jesús Novalbos, Miguel Ángel Seguido, Francisco Abad-Santos, Mar Hernaez, Mercè Brunet, David Hansoe Heredero-Jung, Alejandro de la Sota-Pérez, María Isidoro-García, Almudena Gil-Rodriguez, Alba Barral-Raña, Olalla Maroñas, Gladys Guadalupe Olivera Pasquini, Enrique G. Zucchet, María José Herrero, José Manuel Dodero-Anillo, Alicia Alba Máñez, María José Pedrosa-Martínez, Adrián Llerena, on behalf of the BioFRAM PGx Consortium

**Affiliations:** 1 Instituto Universitario de Investigación Biosanitaria de Extremadura (INUBE), 06080 Badajoz, Spain; francisco.ariasa@externos.salud-juntaex.es (F.A.-A.); mariadelcarmen.mata@salud-juntaex.es (C.M.-M.); albamaria.sanchezr@externos.salud-juntaex.es (A.S.-R.); 2 Pharmacogenetic and Personalised Medicine Unit, Extremadura Health Service, Badajoz University Hospital, Extremadura University, 06080 Badajoz, Spain; 3 Clinical Pharmacology Department, Hospital Universitario de La Princesa, Pharmacology Department, Faculty of Medicine, Instituto de Investigación Sanitaria la Princesa (IIS-Princesa), Universidad Autónoma de Madrid, 28006 Madrid, Spain; jesus.novalbos@salud.madrid.org (J.N.); miguelangel.seguido.externo@salud.madrid.org (M.Á.S.); francisco.abad@uam.es (F.A.-S.); 4 Pharmacology and Toxicology Laboratory, Biochemistry and Molecular Genetics Department, Biomedical Diagnostic Center, Hospital Clinic of Barcelona, University of Barcelona, Fundació Clínic per a la Recerca Biomèdica (FCRB), 08036 Barcelona, Spain; mhernaez@recerca.clinic.cat; 5 Pharmacology and Toxicology Laboratory, Biochemistry and Molecular Genetics Department, Biomedical Diagnostic Center, Hospital Clinic of Barcelona, University of Barcelona, Fundació de Recerca Clínic Barcelona-Institut d’Investigacions Biomèdiques August Pi i Sunyer (FCRB-IDIBAPS), Centro de Investigación Biomédica en Red Enfermedades Hepáticas y Digestivas (CIBERehd), 08036 Barcelona, Spain; mbrunet@clinic.cat; 6 Institute of Biomedical Research of Salamanca (IBSAL), 37007 Salamanca, Spain; dhheredero.ibsal@saludcastillayleon.es (D.H.H.-J.); asota.ibsal@saludcastillayleon.es (A.d.l.S.-P.); misidoro@saludcastillayleon.es (M.I.-G.); 7 Department of Clinical Laboratory, Salamanca University Hospital (HUS), 37007 Salamanca, Spain; 8 Department of Medicine, University of Salamanca, 37007 Salamanca, Spain; 9 Pharmacogenomics and Drug Discovery Group (GenDeM), Health Research Institute of Santiago de Compostela (IDIS), 15706 Santiago de Compostela, Spain; almudena.gil@usc.es (A.G.-R.); alba.barral.rana@gmail.com (A.B.-R.); olalla.maronas@usc.es (O.M.); 10 Genomics and Bioinformatics Group, Centre for Research in Molecular Medicine and Chronic Diseases (CiMUS), University of Santiago de Compostela, 15782 Santiago de Compostela, Spain; 11 Galician Public Foundation of Genomic Medicine (FPGMX), Galician Health Service (SERGAS), 15706 Santiago de Compostela, Spain; 12 Department of Pharmacology, Universitat de València, 46100 Valencia, Spain; gladys_guadalupe@iislafe.es (G.G.O.P.); enrique_zucchet@iislafe.es (E.G.Z.); maria.jose.herrero@uv.es (M.J.H.); 13 Pharmacogenetics and Gene Therapy Unit, Instituto Investigación Sanitaria La Fe (IIS La Fe), 46026 Valencia, Spain; 14 Clinical Pharmacology Unit, Hospital Universitario de Puerto Real, 11510 Puerto Real, Spain; dodero.web@gmail.com (J.M.D.-A.); alicia.alba@inibica.es (A.A.M.); mjose.pedrosa.sspa@juntadeandalucia.es (M.J.P.-M.)

**Keywords:** pharmacogenetics, pharmacogenomics implementation, Spanish National Health System, adverse drug reactions, clinical guidelines, level 1a evidence, multicentre study, personalized medicine

## Abstract

**Background/Objectives:** Pharmacogenetics (PGx) may improve drug safety and effectiveness, but its integration into routine care remains heterogeneous across the Spanish National Health System (NHS). This article presents BioFRAM PGx, a multicentre implementation framework that defines the methodological and operational basis for subsequent observational validation in cardiovascular and mental-health care settings. **Methods:** The framework was developed by healthcare centres from eight Spanish Autonomous Communities through a structured review of PharmGKB/ClinPGx clinical annotations, CPIC and DPWG guidelines, and national regulatory resources. Gene–drug pairs were prioritized according to clinical actionability, therapeutic relevance, applicability to the Spanish population, and analytical feasibility. BioFRAM PGx defines a panel of seven pharmacogenes and 35 drugs, standardized genotyping and phenotype-assignment procedures, pharmacovigilance and clinical variables, healthcare-resource measures, and centralized data management. The proposed non-interventional validation phase includes adults receiving at least one selected drug in hospital or primary-care settings, with a minimum six-month follow-up. PGx results are not returned to clinicians and do not modify treatment. Its primary objective is to assess the predictive value of PGx genotyping for adverse drug reactions (ADRs); secondary objectives include ADR incidence and implementation feasibility. **Results:** The main outputs are the proposed multicentre observational design, the prioritized gene–drug panel, harmonized analytical and pharmacovigilance workflows, standardized data domains, and a centralized data-management strategy. No patient-level clinical, implementation, or economic outcomes are presented. **Conclusions:** BioFRAM PGx provides a structured multicentre framework for harmonizing pharmacogenetic procedures across the Spanish NHS. Its clinical effectiveness, influence on prescribing, scalability, and economic outcomes remain to be assessed in subsequent studies.

## 1. Introduction

Pharmacogenetics (PGx) investigates how an individual’s genetic makeup affects their response to drugs [1,2]. As a tool in personalized medicine, PGx enables the optimization of drug therapy by tailoring treatments to individual genetic profiles. The identification of pharmacogenetic biomarkers (gBMs), defined as genetic variants or genetic characteristics associated with drug response, efficacy, toxicity, or dose requirements, can help prevent adverse drug reactions (ADRs) and therapeutic failures across a wide range of clinical contexts. The Spanish National Health System has recently incorporated pharmacogenetic testing into the national shared portfolio of genetic and genomic services [3]. However, the existence of a national catalogue does not necessarily guarantee uniform regional availability, operational implementation, or routine clinical uptake. As a result, pharmacogenetic services remain heterogeneous across healthcare settings, highlighting the need for harmonized implementation strategies that facilitate equitable access and consistent clinical integration nationwide. 

Within Europe, the European Medicines Agency (EMA) provides PGx information by incorporating relevant guidance into medicinal product labels for both prescribers and patients. This evidence is primarily derived from studies investigating genetic determinants of drug pharmacokinetics and pharmacodynamics [4]. This evidence base is complemented by clinical trials evaluating the impact of genotype-guided dosing approaches on patient outcomes [5]. However, most of the evidence stems from research settings, rather than everyday clinical practice [6]. This reliance on research data, while valuable, presents certain limitations. Research settings often involve highly controlled conditions and selected patient populations, which may not fully reflect the real-world heterogeneity and complexities encountered in routine clinical care. For example, research studies typically have strict inclusion and exclusion criteria that limit participant diversity, such as recruiting patients within a specific age range or those with no comorbidities. Additionally, the treatment protocols in research settings are often highly standardized, whereas in clinical practice, patients may receive a variety of concomitant medications that can influence drug response. Furthermore, factors such as medication adherence, self-medication practices, lifestyle, and socioeconomic status, which can affect drug efficacy and safety, are not always accounted for in the controlled environment of a research study. This gap underscores the need for further studies that can bridge the divide between the carefully controlled research settings and the complex realities of real-world clinical practice. Such studies are essential to validate the true utility and applicability of pharmacogenetics in routine clinical care, where patients present with diverse characteristics, comorbidities, and concomitant medications that may influence drug response. To address this gap at a continental level, the European Commission’s Horizon 2020 program funded the Ubiquitous Pharmacogenomics consortium [7]. This collaborative network, spanning seven European healthcare environments—including Spain, the Netherlands, and the UK—sought to transition PGx from a single drug-gene pair approach to a preemptive, panel-based strategy. A cornerstone of this effort was the Preemptive Pharmacogenomic Testing for Preventing Adverse Drug Reactions study, a large-scale, multicentre trial involving approximately 6944 patients [8]. In the PREPARE study, patients carrying actionable pharmacogenetic variants who received genotype-guided prescribing experienced a lower incidence of clinically relevant adverse drug reactions than those receiving standard care. During the first 12 weeks of follow-up, clinically relevant ADRs occurred in 21.0% of patients in the genotype-guided group and 27.7% of patients in the standard-care group, corresponding to an approximately 30% relative reduction in risk [9]. By utilizing Dutch Pharmacogenomics Working Group guidelines and advanced IT solutions for clinical decision support, the project demonstrated the feasibility and effectiveness of the approach across diverse European healthcare settings [10]. While the U-PGx project established a successful framework for cross-border implementation, its findings emphasize that the successful adoption of PGx requires adapting these models to the specific clinical workflows and regulatory idiosyncrasies of national health systems. Therefore, bridging the gap between international research and local real-world implementation is crucial to validate pharmacogenetics’ utility in everyday practice. 

Building upon the groundwork laid by regional initiatives, such as the MedeA project in Extremadura, which successfully integrated pharmacogenetics and clinical data into electronic health records to support individualized drug prescription [1], this implementation proposal introduces BioFRAM PGx. This model incorporates structural lessons and evidence from other key regional programs across Spain. In Madrid, the “PriME-PGx” initiative at the Hospital Universitario de La Princesa serves as a multidisciplinary model for implementing pharmacogenetics in hospital care [11,12]. In Galicia, the Galician Public Foundation of Genomic Medicine centralizes genetic testing to optimize pharmacological responses [13,14]. Furthermore, the “5sPM” (5 steps in Personalised Medicine) model at the Hospital Universitario de Salamanca provides a structured framework for applying pharmacogenetics to cases of therapeutic failure and adverse reactions [15,16]. Catalonia is also integrated into this collaborative landscape through the clinical and molecular expertise of the Hospital Clínico de Barcelona [17,18]. These individual regional strategies are being harmonized within the BioFRAM PGx national effort, which aims to establish a shared pharmacogenetic implementation framework for the Spanish National Health System, specifically focused on cardiovascular and mental-health care.

The primary objective of this proposal is to develop and describe a standardized implementation framework for the application of a prioritized PGx marker panel within the Spanish National Health System. As a multicentre initiative spanning eight regions, BioFRAM PGx aims to transition from isolated regional approaches towards a harmonized framework for adult patients receiving cardiovascular or mental-health care. To achieve this, the proposal defines the selected pharmacogene–drug panel, analytical and phenotype-assignment procedures, clinical and pharmacovigilance variables, data-management processes, and multicentre organizational structure required for its application across participating healthcare settings. By standardizing these components, BioFRAM PGx establishes the methodological and operational basis for the future evaluation of associations between selected pharmacogenetic genotypes and predicted phenotypes and adverse drug reactions under real-world conditions, including their predictive performance.

This project is expected to contribute to the implementation of pharmacogenetics in routine clinical care by supporting harmonized procedures across different real-world healthcare settings and informing future implementation and evaluation strategies within the Spanish National Health System. Its clinical effectiveness, impact on prescribing decisions, scalability, and economic value remain to be assessed in subsequent studies.

## 2. Results

The Results section presents the principal outputs of the BioFRAM PGx framework-development process. First, we detail the overall study design that structures the proposed model and defines its operational components. This is followed by the presentation of the key elements supporting the implementation strategy, including the selection of clinically relevant drugs, the associated pharmacogenetic biomarkers, and the clinical variables required for future validation. Together, these components constitute the core outputs and foundational elements of the proposal necessary for the subsequent clinical, pharmacovigilance, and economic evaluation of the PGx implementation model.

### 2.1. Framework Design

The BioFRAM PGx implementation proposal is conceptualized as a foundational framework for the systematic and harmonized application and future evaluation of pharmacogenetics across the Spanish NHS. Developed through a multicentre collaboration involving eight Autonomous Communities, the framework defines shared methodological and operational components intended to address the fragmentation that has characterized PGx implementation within Spain’s decentralized healthcare model [19].

BioFRAM PGx is intended to support the generation of standardized real-world pharmacogenetic, clinical, and pharmacovigilance data under routine-care conditions. Pharmacogenetic results are not returned to treating clinicians and do not inform or modify treatment decisions within the framework. Consequently, the present proposal does not evaluate genotype-guided prescribing or clinical effectiveness. Its multicentre structure provides a basis for the subsequent assessment of the framework’s applicability across participating healthcare settings, while its scalability and broader transferability remain to be evaluated. Following development of the BioFRAM PGx framework, a multicentre observational validation study was initiated across participating consortium centres, and recruitment has now been completed. No patient-level data or clinical outcomes from this observational phase are reported in the present article, which focuses exclusively on the development of the BioFRAM PGx implementation framework. Results from the validation study will be analysed and reported separately.

### 2.2. Drug Selection

The 35 drugs included in the BioFRAM PGx framework (Table 1) were selected within the predefined scope of cardiovascular and mental-health care. Relevant therapeutic agents from Anatomical Therapeutic Chemical (ATC) groups B, C, and N were identified using national and regional prescribing information, the clinical experience of the participating groups, the availability of clinically relevant pharmacogenetic evidence and actionable recommendations, and compatibility with the analytical capabilities available within the project. The resulting list represents a selected set of drugs considered relevant for the intended clinical settings and should not be interpreted as an exhaustive catalogue of all drugs with clinically actionable pharmacogenetic associations.

### 2.3. Pharmacogene Selection

The selection of pharmacogenes in the BioFRAM PGx project was based on the cumulative criteria described in Section 4.2. A ClinPGx Level 1A clinical annotation was used as an initial evidence-screening criterion, while the availability of actionable prescribing recommendations from CPIC, DPWG, or relevant regulatory sources was assessed separately [20,21,22]. Additional criteria included the clinical and functional relevance of the pharmacogenetic association, its applicability to the target population, its relevance to cardiovascular and mental-health care, and its compatibility with analytically validated genotyping methods available within the project. The variants and alleles selected for testing were required to have established clinical relevance and documented functional consequences relevant to drug response.

The resulting panel was deliberately selected for the predefined clinical scope and analytical workflow and was not intended to provide exhaustive coverage of all clinically actionable pharmacogenetic associations. Pharmacogenes such as *DPYD*, *TPMT*, *NUDT15*, and *UGT1A1* were not included because their principal actionable applications relate mainly to therapeutic areas outside the cardiovascular and mental-health scope of BioFRAM PGx, including oncology, haematology, immunology, transplantation, and infectious diseases [23,24]. Their exclusion should not be interpreted as indicating limited clinical relevance.

*HLA-A* and *HLA-B* were also considered but were not included in the initial panel. BioFRAM PGx was designed primarily to evaluate pharmacogenetic variability relevant to the optimization of routinely used pharmacotherapy rather than mandatory pre-treatment screening for severe immune-mediated hypersensitivity reactions. In addition, HLA typing was not part of the analytically validated workflow selected for the project. Their exclusion should not be interpreted as a recommendation against clinically or regulatorily indicated *HLA* testing, which remains applicable according to relevant clinical guidelines and authorised medicinal product information [25]. Phenytoin was retained because of its actionable association with *CYP2C9* [26]; however, the pharmacogenetic coverage provided by BioFRAM PGx does not replace *HLA* testing when this is clinically indicated.

Other genes were considered but were not included in the initial panel. *CYP2B6* was evaluated during the selection process; however, the panel was completed in December 2022, before publication of the 2023 CPIC guideline update introducing *CYP2B6*-based recommendations for sertraline [27]. *CYP4F2* was also considered for warfarin dose prediction, but its contribution was regarded as incremental relative to the combined contribution of *CYP2C9* and *VKORC1* within the predefined scope of the framework [28]. These exclusions reflect the clinical scope, evidence available at the review cut-off, and analytical feasibility of the initial panel rather than a lack of clinical importance. Low allele frequency alone was not used as a sufficient criterion for excluding clinically relevant variants.

Analytical feasibility was an additional requirement for inclusion. The selected pharmacogenes and variants had to be compatible with validated analytical methods available within the participating laboratories [29,30]. This criterion does not imply complete detection of all rare or complex structural variants, and the analytical coverage and limitations of the methods used must be documented, particularly for structurally complex loci such as *CYP2D6*.

As a result of this process, the BioFRAM PGx framework includes seven pharmacogenes and their corresponding selected variants and alleles (Table 2). The associated pharmacogene–drug relationships are summarized in Table 2, while the complete list of targeted genetic variants and their genomic and functional characteristics is provided in Table S1–S4. The current panel should be regarded as an initial selected version that will require future reassessment through multidisciplinary expert review when clinically relevant changes arise in prescribing guidelines, regulatory requirements, allele-definition or phenotype-translation frameworks, analytical technologies, or relevant allele-selection standardization frameworks, including AMP recommendations where applicable.

### 2.4. Clinical Variables Selection

The selection of clinical variables for the BioFRAM PGx framework was based primarily on a review of the CPIC and DPWG guidelines corresponding to the selected pharmacogene–drug relationships [20,21,22,26,31,32]. These guidelines were used to identify the clinically relevant drug-response and safety domains associated with variation in genotype or predicted phenotype, including altered drug exposure, dose requirements, reduced therapeutic response, and increased risk of specific adverse drug reactions where described. The clinical consequences and monitoring parameters identified in these sources were translated into operational variables suitable for harmonized collection across participating centres. This guideline-based selection was complemented by expert clinical and pharmacovigilance assessment and by variables required to characterize treatment exposure, concomitant medication, physiological status, and other factors that could influence the observed drug response.

The resulting variable set combines a common core dataset applicable across drugs and healthcare settings with drug- and therapeutic-group-specific outcome domains. These variables are intended to support subsequent observational analyses of associations between selected pharmacogenetic genotypes or predicted phenotypes, drug exposure, ADRs, and other clinically relevant outcomes under real-world conditions. They do not constitute predefined clinical-effectiveness endpoints, because pharmacogenetic results are not returned to treating clinicians and do not modify treatment within the present framework.

Drug-specific variables reflect the clinical consequences associated with the selected pharmacogene–drug relationships. These include point international normalized ratio (INR) values and bleeding or thromboembolic events for vitamin K antagonists; lipid response, creatine kinase, and muscle symptoms for statins; relevant cardiovascular outcomes; neurological tolerability and treatment-pattern variables for phenytoin; pain-related status and treatment-pattern variables for tramadol; and selected UKU (Udvalg for Kliniske Undersøgelser) adverse-effect items for psychotropic drugs. Time in therapeutic range, the complete UKU scale, and validated psychiatric efficacy scales are not systematically available.

The modified Karch–Lasagna algorithm is incorporated as a structured pharmacovigilance method for evaluating and classifying the likelihood of a causal relationship between a drug and an adverse event [33,34]. It assesses criteria including temporal sequence and alternative explanations and classifies the drug–event relationship into categories ranging from “unlikely” to “definite”. This assessment does not determine whether a pharmacogenetic factor contributed to the event. Evaluation of a potential pharmacogenetic contribution requires integration of the drug–event causality assessment with genotype or predicted-phenotype information, the corresponding pharmacogene–drug evidence, treatment exposure, concomitant medications, and other relevant clinical variables.

Healthcare-resource utilization variables, including emergency department visits, hospitalizations and their duration and reason, and specialist referrals, are collected as an exploratory component intended to inform future economic studies [35,36]. The present framework does not perform a formal cost-effectiveness evaluation. Physiological and clinical variables, including renal and hepatic function, concomitant treatments, and lifestyle factors, allow potential confounding and phenoconversion to be considered in subsequent analyses [37,38]. A summarized overview of the common clinical-variable domains is presented in Table 3. Supplementary Table S5–S8 provide the complete variable definitions, while Table S9 maps the selected drugs and therapeutic groups to the corresponding outcome domains and variables available within the BioFRAM PGx framework.

### 2.5. Proposed Data Collection and Management Strategy

The BioFRAM PGx framework includes a standardized multicentre data collection and management strategy intended to support the harmonized collection of pharmacogenetic, clinical, pharmacovigilance, and healthcare-resource data during its subsequent observational application. All data collection, handling, and storage procedures will be conducted in accordance with the World Medical Association (WMA) Declaration of Helsinki for medical research involving human subjects [39] and applicable Spanish regulations, including Royal Decree 957/2020 and the Organic Law on Data Protection (LOPDGDD 3/2018).

#### Integration Workflow

**Figure 1:**
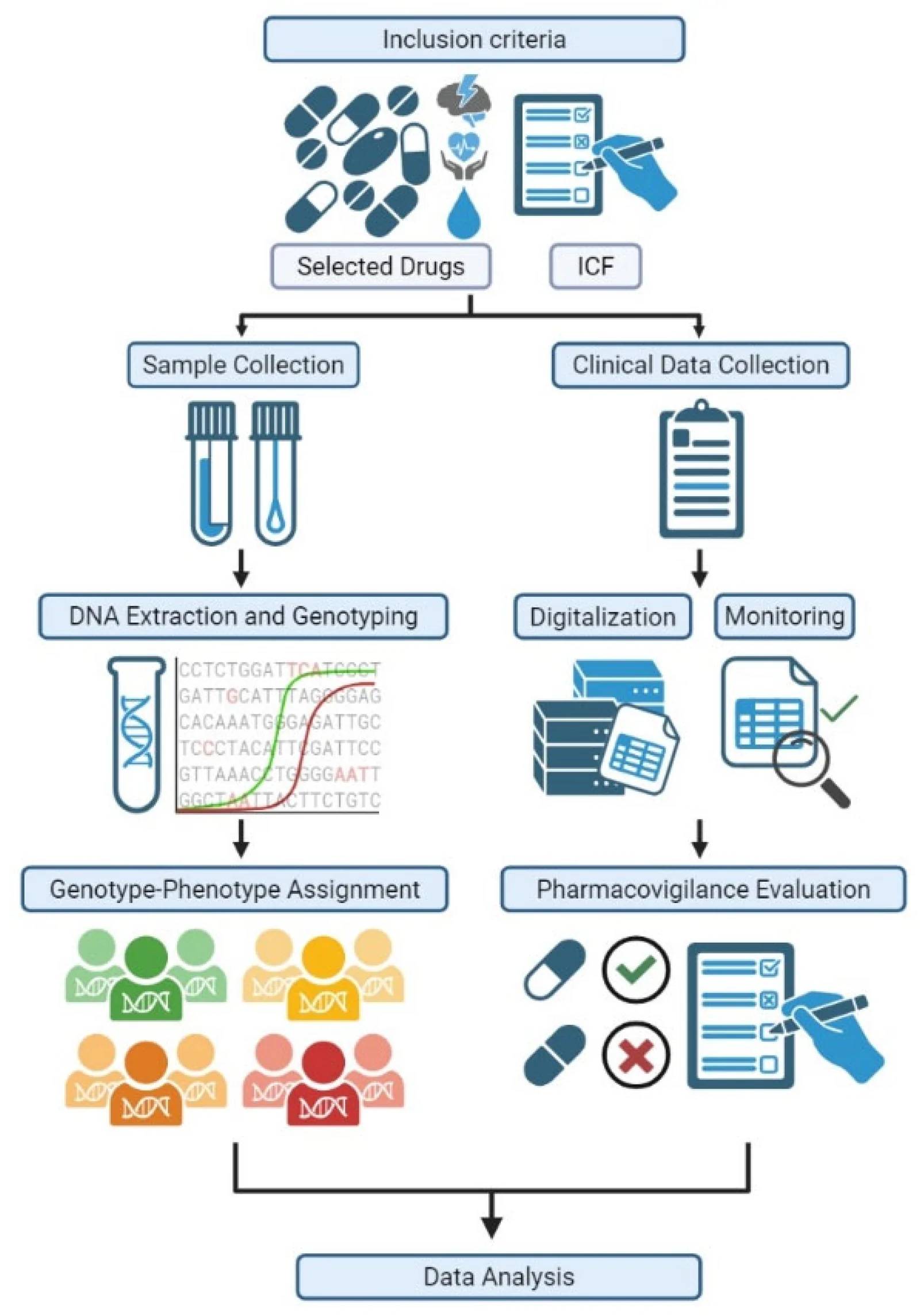
Schematic representation of the BioFRAM PGx proposed observational, analytical, and data-management workflow. Created in BioRender. Arias Aragón, F. (2026) https://BioRender.com/28rx30f (accessed on 31 July 2026).

The proposed data collection process comprises a six-phase workflow (Figure 1) designed to standardize data capture, traceability, and quality control across participating centres:

Patient Enrolment and Pseudonymization: During application of the framework, written informed consent will be obtained before data and biological samples are collected. Patient information—including identification data, sample-collection date, originating centre, sample type, responsible healthcare provider, laboratory reception date, and laboratory code—will be pseudonymized using a standardized alphanumeric coding system (e.g., “0-XXX-000”). Direct identifiers and linkage information will be stored separately from the research dataset, with access restricted to authorized personnel at each recruiting centre.

Digitalization via REDCap: To support secure multicentre collaboration, the framework uses the Research Electronic Data Capture platform (REDCap) [40,41]. This metadata-driven methodology allows for the seamless transition of paper-based clinical records into a secure, password-protected electronic Case Report Form (eCRF), providing a centralized repository for both clinical and genetic variables.

Standardized Pharmacovigilance Assessment: The framework incorporates the modified Karch–Lasagna algorithm as a structured method for assessing the likelihood of a causal relationship between a drug and an adverse event [33,34]. This assessment concerns drug–event causality and does not establish whether a pharmacogenetic factor contributed to the event.

Healthcare Resource Utilization Analysis: Emergency department visits, hospitalizations—including their duration and reason—and specialist referrals will be documented during the predefined retrospective and prospective observation periods. These data constitute an exploratory component intended to inform future economic studies and do not represent a formal cost-effectiveness evaluation within the present framework [35,36].

Quality Assurance and Monitoring: To maintain the proposal’s rigor, a dedicated monitoring officer (independent from the data entry personnel) will perform regular audits. This phase is designed to resolve queries and ensure that the digitalized data in REDCap provides a faithful representation of the source clinical documents.

Database Closure and Readiness for Analysis: Following completion of data-quality checks and resolution of outstanding queries, the database will be formally locked. The resulting dataset will be prepared for subsequent observational analyses of associations between pharmacogenetic genotypes or predicted phenotypes, drug exposure, ADRs, and other relevant clinical outcomes.

Together, these components define a standardized and secure multicentre workflow for pseudonymized data capture, structured pharmacovigilance assessment, documentation of healthcare-resource utilization, and quality-controlled database closure. The strategy is intended to generate harmonized data suitable for the subsequent evaluation of pharmacogenetic associations and their predictive performance, rather than to demonstrate clinical effectiveness, safety benefits, implementation success, or economic impact. Its future application will remain subject to the applicable ethical, regulatory, data-protection, and quality requirements.

### 2.6. Sample Processing and Analysis

#### Proposed Methodological Framework for Biological Sample Collection and Processing

As part of the proposed BioFRAM PGx methodological framework, this section defines common principles and minimum documentation requirements for biological sample collection and processing across participating regions of the Spanish NHS. During future application of the framework and following the verification of the eligibility criteria and acquisition of written informed consent, a blood or saliva sample would be collected from each patient [42]. While blood remains a primary source for genomic DNA, saliva is included as a non-invasive alternative that may facilitate patient participation and sample collection in routine-care settings [43,44,45].

Samples would be stored and transported under conditions appropriate to the sample type and according to validated procedures at each participating laboratory. Genomic DNA would be isolated using validated extraction methods and standardized commercial kits optimized for whole blood and stabilized saliva samples [46,47]. To support harmonization and traceability, each laboratory would document the sample type, collection and storage conditions, extraction method, DNA concentration and quality, and predefined acceptance criteria. Procedures would follow validated laboratory protocols and, where applicable, manufacturer instructions. This approach is intended to provide genomic DNA of sufficient quality for the analysis of the selected variants and, where supported by the assay, copy-number variation. The analytical coverage and limitations of the methods used would be documented as described in the following section.

### 2.7. Proposed Pharmacogenetic Analysis and Predicted Phenotype Assignment

As part of the proposed BioFRAM PGx methodological framework, pharmacogenetic testing would follow a decentralized and platform-independent approach. Analyses would be performed locally by participating centres or their associated validated laboratories rather than in a single central laboratory. Because different validated analytical systems may be used, the framework does not prescribe a single commercial assay or universal genotype-calling threshold. Each laboratory would document the testing site, analytical platform or assay, targeted variants, structural-variant coverage, validated genotype-calling criteria, quality-control requirements, repeat-testing procedures, and relevant analytical limitations.

Testing would be performed using locally validated molecular genotyping methods suitable for the detection of the selected variants. Depending on the participating laboratory, these may include allele-specific, hybridisation-based, primer extension, or sequencing-based approaches—capable of achieving precise discrimination between wild-type and variant sequences [30,48,49]. Internal quality-control samples and appropriate positive and negative controls would be included according to local validation procedures. Ambiguous, failed, or discordant results would undergo repeat analysis when technically feasible. Results remaining unresolved after repeat testing would be classified as indeterminate and would not receive a definitive diplotype or predicted-phenotype assignment.

The analytical resolution of structural variation would depend on the platform and assay used. In particular, targeted PCR- or qPCR-based approaches may not fully resolve allele-specific *CYP2D6* duplications or multiplications, *CYP2D6/CYP2D7* hybrid alleles, tandem arrangements, or other complex structural configurations. Consequently, some *CYP2D6* diplotypes may remain ambiguous or partially resolved and may require orthogonal or additional analyses beyond the initial analytical workflow. Results would therefore be reported according to the analytical resolution achieved, and a definitive predicted phenotype would only be assigned when the genotype or diplotype can be interpreted reliably.

Once the analytical phase confirms the presence of specific genetic variants, the implementation proposal transitions from laboratory detection to the systematic translation of genomic data into standardized predicted phenotypes or functional categories. This interpretive process serves as the essential bridge between raw diplotype information and the standardized metabolic categories (such as poor, intermediate, normal, or ultrarapid metabolizers) for subsequent observational analyses. To ensure that genetic results are interpreted consistently and remain comparable across the diverse clinical settings of the Spanish NHS, phenotype assignment relies on harmonized terminology and curated genotype-to-phenotype translation tables provided by international consensus resources. Allele definitions and nomenclature would follow PharmVar where applicable, while phenotype assignment would follow the relevant CPIC and DPWG consensus recommendations and gene-specific translation tables [32,50]. The following section describes the proposed framework for assigning functional activity scores and predicted metabolic phenotypes from interpretable genetic profiles:

For the *CYP2D6* and *CYP2C9* genes, an estimated metabolic phenotype is assigned based on their corresponding enzymatic activity. The phenotype classifications include:

- *CYP2D6*: Poor metabolizer (PM, no function), intermediate metabolizer (IM, decreased function), normal metabolizer (NM, normal function), and ultrarapid metabolizer (UM, increased function).
- *CYP2C9*: Poor metabolizer (PM, no function), intermediate metabolizer (IM, decreased function), and normal metabolizer (NM, normal function).

For activity-score calculation, each interpretable allele would be assigned a functional value according to the applicable translation recommendations, as summarized in Table 4.

**Figure 2:**
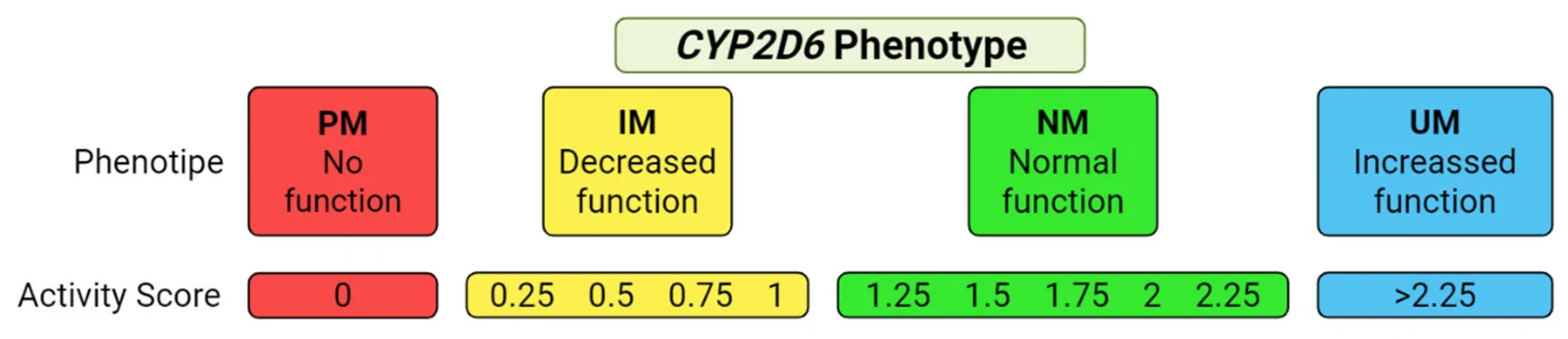
Translation of CYP2D6 activity scores into predicted metabolizer phenotypes. The CYP2D6 phenotype is determined by the total Activity Score (AS) of the alleles. Individuals are classified into four categories: Poor Metabolizers (PM) with no enzymatic function (AS = 0); Intermediate Metabolizers (IM) with decreased function (AS = 0.25–1); Normal Metabolizers (NM) with normal function (AS = 1.25–2.25); and Ultrarapid Metabolizers (UM) with increased function (AS > 2.25). Created in BioRender. Arias Aragón, F. (2026) https://BioRender.com/xqhnwkj (accessed on 31 July 2026).

**Figure 3:**
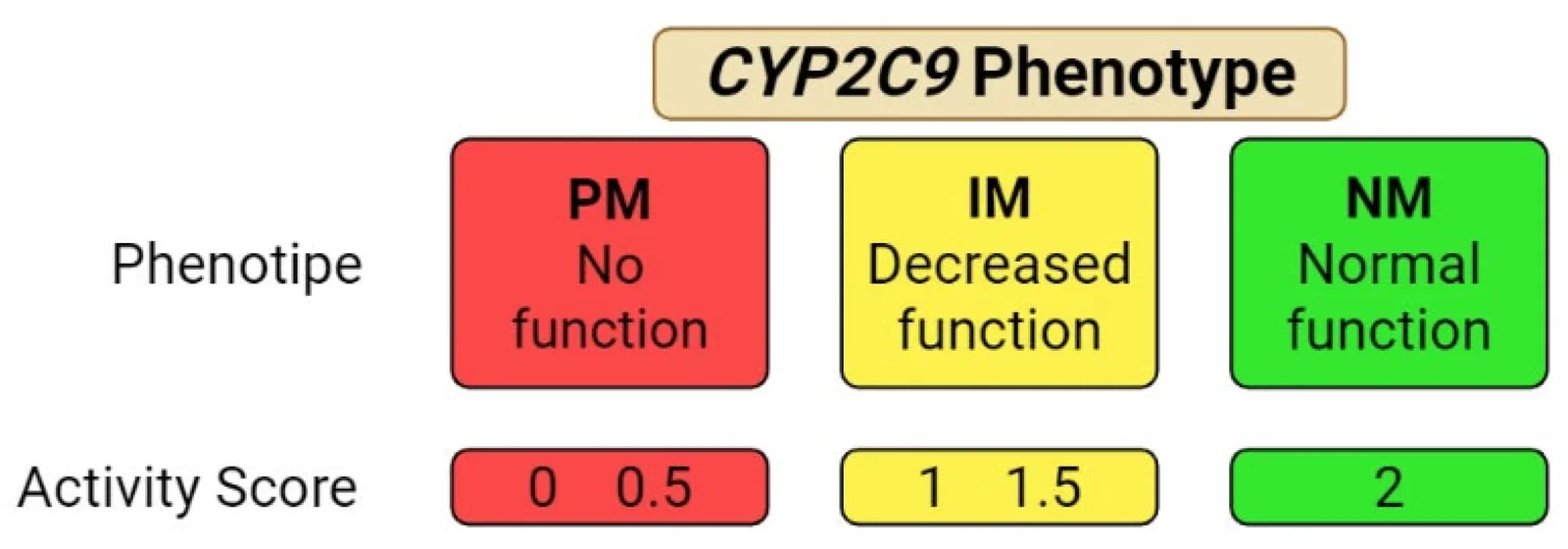
Translation of CYP2C9 activity scores into predicted metabolizer phenotypes. The CYP2C9 predicted phenotype is assigned according to the total Activity Score (AS), calculated from the functional values of the interpretable alleles. Activity scores of 0–0.5 are classified as poor metabolizer (PM), scores of 1–1.5 as intermediate metabolizer (IM), and a score of 2 as normal metabolizer (NM), according to the applicable genotype-to-phenotype translation framework. Created in BioRender. Arias Aragón, F. (2026) https://BioRender.com/6hlwxj5 (accessed on 31 July 2026).

The predicted phenotype is assigned from the total activity score obtained by summing the values attributed to each interpretable allele. For *CYP2D6*, activity scores of 0, 0.25–1, 1.25–2.25, and >2.25 correspond to PM, IM, NM, and UM categories, respectively [51] (Figure 2). For *CYP2C9*, scores of 0–0.5, 1–1.5, and 2 correspond to PM, IM, and NM categories, respectively [26] (Figure 3). These assignments are made according to the documented translation source and version used for the analysis.

For the remaining genes included in the panel (*CYP2C19*, *CYP3A4*, *SLCO1B1*, *VKORC1*, and *ABCG2*), predicted phenotypes or functional categories would be assigned by mapping interpretable genotypes or diplotypes using standardized gene-specific translation resources and the applicable CPIC or DPWG recommendations, where available. [48,52]. For *SLCO1B1* and *ABCG2*, interpretation would describe transporter-function categories, whereas *VKORC1* would be classified according to the relevant expression or vitamin K antagonist sensitivity category [53,54].

Predicted-phenotype assignments within BioFRAM PGx are intended for standardized pharmacogenetic classification of genotype/phenotype–drug–ADR associations. They are not returned to treating clinicians and are not used to guide or modify treatment within the present framework.

### 2.8. Proposed Data Analysis Framework

Consistent with the methodological scope of the present proposal, this section outlines general principles for the future analysis of data generated through application of the BioFRAM PGx framework rather than a prespecified statistical analysis protocol. The specific analytical methods and model specifications will depend on the characteristics, distribution, and availability of the data generated during its application.

Quantitative variables will be summarized using appropriate measures of central tendency, dispersion, and distribution, while categorical variables will be described using frequencies and proportions. Associations between variables may be explored using appropriate measures according to variable type, including Cramér’s V and Pearson or Spearman correlation coefficients. The proportions of ADRs across different genotypes and metabolic phenotypes will be compared. Where supported by the available sample size and number of events, the ability of pharmacogenetic biomarkers to predict ADRs will be explored using sensitivity, specificity, predictive values, likelihood ratios, and other relevant performance measures.

Associations between genetic factors, predicted phenotypes, drug exposure, and clinical or pharmacovigilance outcomes will be assessed using appropriate univariate and multivariable methods. Depending on the type and distribution of the outcome, comparisons may include Student’s *t*-test or ANOVA and their corresponding non-parametric alternatives, while logistic or linear regression models may be used to estimate adjusted associations. Multivariable models will incorporate clinically relevant prespecified covariates rather than selecting variables solely according to statistical significance in univariate analyses. Potential multicollinearity will be assessed before model fitting.

The analytical approach will also account, where applicable, for the multicentre structure of the data, exposure to multiple selected drugs, repeated measurements within individuals, and relevant clinical confounding. Missing data, multiple comparisons, and sparse pharmacogene–drug–outcome combinations will be evaluated and reported explicitly, and analyses with limited numbers of observations or events will be interpreted as exploratory. Healthcare-resource utilization data will be analysed descriptively and exploratorily to inform future health-economic evaluations. Formal cost-effectiveness analyses, including comparator selection, costing methodology, analytical perspective, time horizon, outcome measures, and uncertainty analyses, fall beyond the scope of the present framework and will be defined in subsequent dedicated studies.

## 3. Discussion

BioFRAM PGx was developed as a multicentre pharmacogenetic implementation framework intended to support the harmonized application and future evaluation of pharmacogenetics in routine-care settings within the Spanish NHS. By involving healthcare institutions from multiple Autonomous Communities, the proposal establishes common methodological and operational criteria for pharmacogene–drug selection, analytical procedures, predicted-phenotype assignment, clinical and pharmacovigilance data collection, and data management. In this context, the primary contribution of BioFRAM PGx is not the demonstration of clinical effectiveness, but the definition of a structured framework that may support subsequent observational, implementation, and validation studies across different healthcare settings.

One of the main strengths of the framework is the selection of pharmacogene–drug relationships supported by ClinPGx Level 1A clinical annotations and actionable recommendations from major pharmacogenetic guidelines or relevant regulatory sources. Rather than aiming to compile all clinically relevant pharmacogenetic associations, the panel was deliberately restricted to genes and drugs considered relevant to the predefined cardiovascular and mental-health scope and compatible with the analytical capabilities available within the project. This selected and non-exhaustive approach was intended to provide a manageable basis for harmonized application across participating centres. Initial analytical and clinical validation is planned within the participating BioFRAM PGx network; no formal independent external-validation cohort is currently planned. Evaluation in non-participating settings would represent a subsequent step for assessing transferability. The December 2022 evidence cut-off also provides a defined temporal reference for the initial panel.

BioFRAM PGx can be considered within the broader European context of pharmacogenetic implementation initiatives such as the Ubiquitous Pharmacogenomics consortium, which demonstrated through the PREPARE study the potential of panel-based genotype-guided prescribing to reduce clinically relevant ADRs in an interventional implementation setting [7,8]. However, BioFRAM PGx addresses a different stage of implementation: pharmacogenetic results generated within the present framework are not returned to clinicians and do not modify treatment. The framework therefore focuses on establishing harmonized procedures and generating standardized observational data rather than evaluating genotype-guided prescribing or its clinical effectiveness. The added value of BioFRAM PGx lies in providing a Spanish-NHS-specific multicentre framework that combines decentralized validated pharmacogenetic testing, standardized phenotype translation, structured pharmacovigilance and harmonized clinical data collection across hospital and primary-care settings.

Another feature of the framework is the integration of harmonized procedures for decentralized genotyping, predicted-phenotype assignment, pharmacovigilance assessment, clinical data collection, and healthcare-resource utilization. Standardized collection of concomitant treatments, renal and hepatic function, and other clinical variables may also allow relevant confounding factors and potential phenoconversion to be considered in subsequent analyses [55,56,57]. Healthcare-resource utilization is included as an exploratory data domain to inform future economic studies rather than as evidence of cost-effectiveness within the current framework [58,59].

The multicentre nature of the initiative represents an additional strength. Participating institutions contribute experience from different healthcare environments and organizational structures, including hospital and primary-care settings within the Spanish National Health System. Although the participating regions were not selected to achieve demographic or population representativeness, their involvement provides a basis for evaluating the applicability of common methodological procedures across heterogeneous clinical settings. Whether the framework is scalable or transferable beyond the participating settings remains to be established.

Several limitations should be acknowledged. First, the present article describes the BioFRAM PGx framework and does not report patient-level outcomes. Its future patient-based application is observational and non-interventional, without a randomized comparator, and pharmacogenetic results are not returned to treating clinicians or used to modify treatment. Consequently, the present framework cannot evaluate the clinical effectiveness of genotype-guided prescribing, prescribing changes, clinician acceptance or override, implementation success, or the effect of clinical decision support.

Second, the population defined for future application consists of patients exposed to at least one of the selected drugs and recruited through participating healthcare centres. It should therefore not be considered representative of the general Spanish population for estimating pharmacogenetic variant frequencies, and geographical origin or recruiting region should not be interpreted as a proxy for genetic ancestry. Importantly, eligibility is independent of ADR status: patients may be included with or without current or previous ADRs, and recruitment is not intentionally enriched for ADR cases. Estimates of ADR occurrence should nevertheless be interpreted within the characteristics of the recruited treated population, patterns of drug exposure, and follow-up. The framework is also not designed to compare reactive and pre-emptive pharmacogenetic testing strategies.

Third, the selected pharmacogenetic panel intentionally includes a limited number of pharmacogenes, alleles, and drug relationships and should not be interpreted as an exhaustive catalogue of clinically actionable pharmacogenetics. Exclusion of particular genes reflects the predefined therapeutic scope, the evidence available at the December 2022 cut-off, and analytical considerations rather than a judgement regarding their clinical relevance. In particular, exclusion of *HLA-A* and *HLA-B* does not replace or contradict clinically or regulatorily indicated *HLA* testing. For phenytoin, the *CYP2C9*-based component of BioFRAM PGx therefore represents partial pharmacogenetic coverage and does not substitute *HLA* testing when clinically indicated.

Fourth, analytical limitations remain inherent to decentralized targeted genotyping. Assay coverage may differ between participating validated laboratories and must therefore be documented together with method-specific limitations. In particular, the structural complexity of the *CYP2D6* locus may limit complete resolution of allele-specific duplications or multiplications, *CYP2D6/CYP2D7* hybrid alleles, tandem arrangements, and other complex structural configurations. Some samples may consequently generate ambiguous or partially resolved diplotypes and require repeat or additional testing; unresolved results are considered indeterminate rather than assigned a definitive predicted phenotype.

Fifth, no formal sample-size calculation or finalized statistical analysis plan is presented in this framework paper, because these will depend on the objectives, exposure distributions, outcome frequencies, and data structure of subsequent patient-level analyses. Rare pharmacogene–drug–event combinations may provide limited statistical power, while the multicentre structure, multiple medications per participant, repeated observations, residual confounding, missing data, and multiple testing will require appropriate consideration. The depth of some drug-specific outcomes is also limited; consequently, analyses unsupported by sufficient observations or events should be regarded as exploratory. 

Sixth, healthcare-resource utilization is collected to inform future health-economic evaluation but does not constitute a formal economic analysis. Cost-effectiveness will require a dedicated study-specific design defining the comparator, analytical perspective, testing and implementation costs, time horizon, outcome measures, and uncertainty analyses. 

Finally, pharmacogenetic knowledge continues to evolve. New clinical guidelines, regulatory recommendations, allele definitions, genotype-to-phenotype translation systems, and analytical technologies may become available after the December 2022 panel-selection cut-off. Future reassessment and updating of the framework will therefore be necessary to maintain alignment with current evidence, without implying a predefined update schedule.

The next stage for BioFRAM PGx is the observational application and evaluation of the proposed framework using harmonized pharmacogenetic, clinical, and pharmacovigilance data. These studies will be required to assess pharmacogene–drug–ADR associations and their predictive performance, as well as the practical applicability of the framework across participating healthcare settings. Dedicated studies will also be required to evaluate broader implementation, clinical effectiveness, transferability, and economic outcomes.

Future clinical implementation of pharmacogenetics would require components that are deliberately outside the scope of the present framework, including effective communication of pharmacogenetic results to healthcare professionals, integration with interoperable electronic health records, clinical decision support, and evaluation of how pharmacogenetic information influences prescribing decisions and patient outcomes. Future revisions may also consider additional pharmacogenes, expanded allele coverage, or alternative analytical technologies as evidence, regulatory requirements, allele definitions, and technical capabilities evolve.

## 4. Materials and Methods

### 4.1. Framework Scope and Objectives

The primary objective of this work is to develop and describe a pharmacogenetic implementation framework for adult patients (≥18 years) receiving cardiovascular or mental-health care within the Spanish NHS. The framework incorporates a panel of 35 clinically relevant drugs and seven pharmacogenes selected through a structured process considering ClinPGx Level 1A clinical annotations, actionable recommendations from major pharmacogenetic guidelines and relevant regulatory sources, therapeutic relevance, applicability to the target population, and analytical feasibility. It also includes a predefined set of clinical and pharmacovigilance variables for the standardized assessment of ADRs.

The framework also aims to establish the methodological and operational basis required for a future observational evaluation of associations between selected pharmacogenetic genotypes and predicted phenotypes, drug exposure, and ADRs under real-world conditions, including their predictive performance. To support this future evaluation, it includes procedures for data collection, patient data entry, digitalization of study records, pharmacovigilance assessment, healthcare resource utilization, monitoring processes, and biological sample processing and analysis. Together, these components are intended to generate harmonized and comparable data across participating healthcare settings.

**Figure 4:**
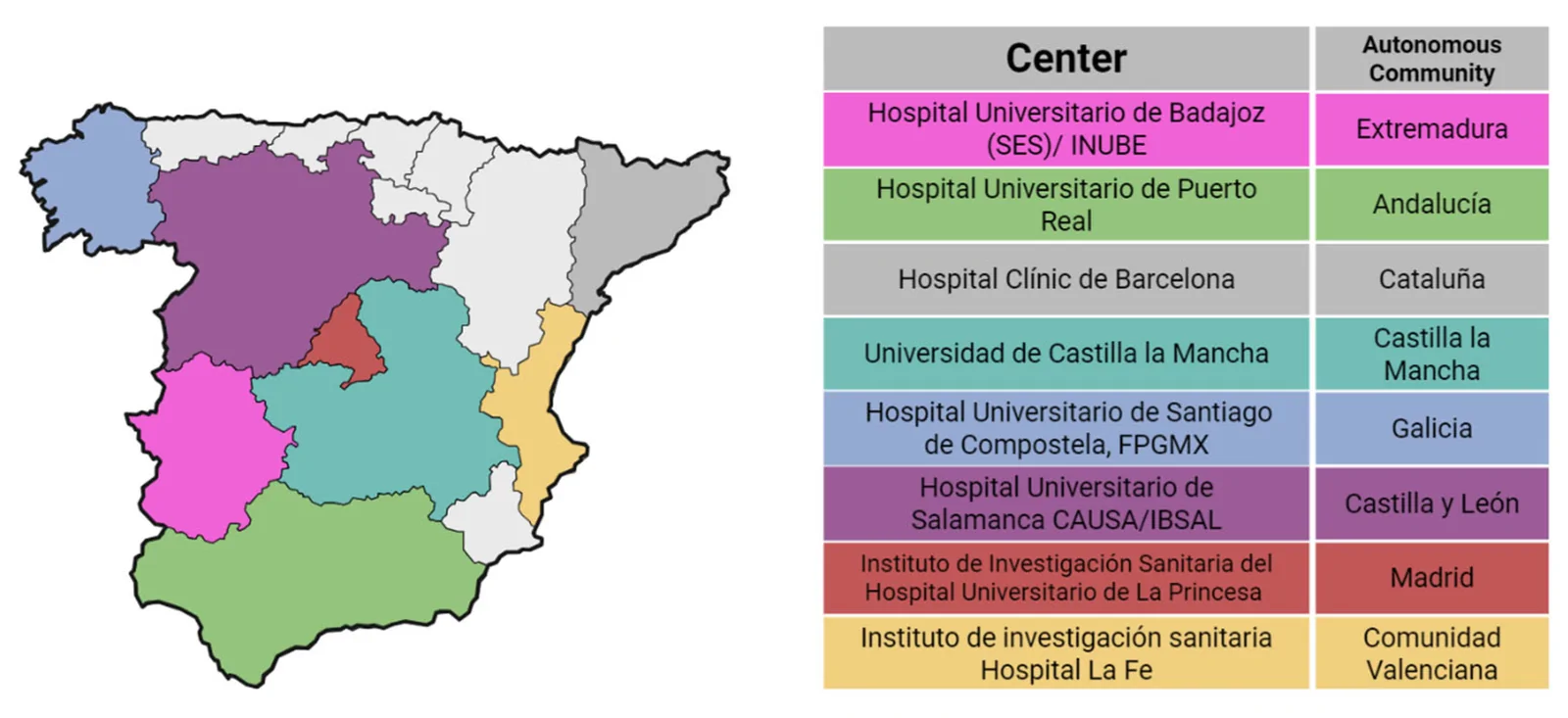
Distribution of the research groups involved in the project. On the left, a map of Spain highlighting the Autonomous Communities participating in the project. On the right, a table listing the Research Centers along with their corresponding Autonomous Communities. Created in BioRender. Arias Aragón, F. (2026) https://BioRender.com/hic12qp (accessed on 31 July 2026).

Importantly, this implementation proposal emerges from a coordinated effort across eight Autonomous Communities in Spain (Figure 4), encompassing different healthcare settings and clinical pathways within the NHS. By integrating contributions from multiple regions, the proposal establishes a shared methodological and operational basis for the harmonized application and future evaluation of the pharmacogenetic panel across participating settings.

### 4.2. Review of Pharmacogenetic Guidelines and Regulatory Databases Supporting the Implementation Proposal

The selection of gBMs and therapeutic agents within the BioFRAM PGx proposal was based on a review of international clinical guidelines and national regulatory databases. The review and panel-selection process were completed in December 2022 and therefore reflect the evidence, recommendations, and regulatory information available at that time. The clinical scope was predefined around adult patients receiving cardiovascular or mental-health care. Within this scope, relevant drugs from ATC groups B (blood and blood-forming organs), C (cardiovascular system), and N (nervous system) were identified using national and regional prescribing information and the clinical experience of the participating groups.

The Clinical Pharmacogenetics Implementation Consortium (CPIC) and the Dutch Pharmacogenetics Working Group (DPWG) are among the most widely used international sources of pharmacogenetic prescribing recommendations and have contributed substantially to the clinical implementation of pharmacogenetics [31,50,60]. Although both organizations provide evidence-based guidance, they use distinct evidence-evaluation and recommendation frameworks, and their recommendations may differ in some clinical situations [50]. These resources are complemented by other national and international guideline initiatives and regulatory sources. In Spain, the pharmacogenomic biomarker database maintained by the Spanish Agency of Medicines and Medical Products (AEMPS) provides structured information on pharmacogenomic biomarkers included in authorised medicinal product labels, facilitating the regulatory integration of pharmacogenetic information into clinical practice [61,62].

Building upon this regulatory and guideline-based foundation, the BioFRAM PGx proposal used the Pharmacogenomics Knowledgebase/ClinPGx [63] as the primary resource for identifying and synthesizing clinically relevant pharmacogenetic associations. ClinPGx Level 1A clinical annotations are generally supported by major pharmacogenetic guidelines or by implementation within a major health system [63,64]. This classification is distinct from the evidence and recommendation systems used by CPIC and DPWG and from regulatory information provided by AEMPS.

After defining the therapeutic scope and identifying the relevant drugs, candidate pharmacogene–drug relationships were assessed according to a set of cumulative criteria. A ClinPGx Level 1A clinical annotation was used as an initial evidence-screening criterion. Because Level 1A annotations may be supported either by major pharmacogenetic guidelines or by implementation within a major health system, the availability of actionable prescribing recommendations in CPIC, DPWG, or relevant regulatory sources was evaluated separately. Additional criteria included the clinical and functional relevance of the genetic association, applicability to the target population, relevance to cardiovascular and mental-health care, prescribing relevance within the Spanish NHS, and compatibility with analytically validated genotyping methods available within the project. The variants or alleles selected for testing were required to have established clinical relevance and documented functional consequences relevant to drug response, including effects on protein function, expression, activity, or transport. 

The resulting proposal was reviewed and discussed by a multidisciplinary group of pharmacogenetics experts from the participating centres, including clinical pharmacologists, geneticists, laboratory specialists, and professionals with experience in the clinical areas covered by the framework. The panel was discussed through telematic consensus meetings. No formal voting procedure was used; the final selection was established by the coordinating group after considering the contributions of the participating experts. Accordingly, the BioFRAM PGx panel represents a selected and analytically feasible set of pharmacogenetic associations tailored to the defined clinical scope, rather than an exhaustive catalogue of all clinically actionable associations.

### 4.3. Target Population

The BioFRAM PGx framework is intended for a real-world clinical population within the Spanish NHS, specifically adults receiving cardiovascular or mental-health care and exposed to at least one of the selected drugs. Its application may involve patients managed in hospital or primary-care settings and at different stages of treatment. This population provides the clinical context for the proposed application and subsequent observational evaluation of the framework.

The framework defines general eligibility parameters to guide future application. Importantly, eligibility is independent of ADR status: patients may be included with no history of ADRs, with a current ADR, or with a previously documented ADR. The proposed recruitment strategy therefore does not intentionally select or enrich the population according to the presence or absence of ADRs. The proposed observational application includes a minimum prospective follow-up of six months after enrolment, complemented by retrospective clinical information where available.

Inclusion Criteria: Patients aged 18 years or older who are currently receiving, or have documented previous exposure to, at least one of the selected drugs and remain under routine clinical follow-up. Participants may be recruited in hospital or primary-care settings and must provide written informed consent before participation.

Exclusion criteria: Patients younger than 18 years; patients with no current or previous exposure to any of the selected drugs; or patients for whom appropriate clinical follow-up is not possible.

Because eligibility is defined within a selected treated clinical population and recruitment is conducted through participating healthcare centres, the resulting population should not be considered representative of the general Spanish population for estimating pharmacogenetic variant frequencies. Similarly, estimates of ADR occurrence should be interpreted within the characteristics of the recruited population, its patterns of drug exposure, and the corresponding follow-up. The framework is not designed to compare reactive and pre-emptive pharmacogenetic testing strategies.

## 5. Conclusions

BioFRAM PGx proposes a multicentre pharmacogenetic implementation framework for the Spanish National Health System. Developed through collaboration across multiple Autonomous Communities, the framework defines a harmonized methodological and operational basis for pharmacogene–drug selection, decentralized analytical procedures, predicted-phenotype assignment, clinical and pharmacovigilance data collection, and data management. The proposal provides a methodological basis for subsequent observational evaluation and future pharmacogenetic implementation initiatives but does not itself demonstrate clinical effectiveness, prescribing impact, implementation success, scalability, reduction in adverse drug reactions, sustainability, or economic value. Dedicated analytical, observational, implementation, clinical-effectiveness, and health-economic studies will be required to evaluate the predictive performance, feasibility, clinical impact, transferability, and economic implications of the framework in routine healthcare settings.

## Figures and Tables

**Table 1:** Selected drugs included in the BioFRAM PGx framework and used to define drug-related eligibility, categorized according to the ATC classification system. Colours indicate the main ATC group: pink, blood and blood-forming organs (B); yellow, cardiovascular system (C); and blue, nervous system (N). Drugs were selected according to their relevance to cardiovascular and mental-health care, clinically relevant pharmacogenetic associations, prescribing relevance within the Spanish National Health System, and compatibility with the analytical capabilities available within the project.

Selected Drugs:					
Clopidogrel	Fluvastatin	Escitalopram	Quetiapine	Nortriptyline	Vortioxetine
Acenocoumarol	Lovastatin	Amitriptyline	Atomoxetine	Zuclopenthixol	
Warfarin	Pitavastatin	Sertraline	Citalopram	Doxepin	
Atorvastatin	Flecainide	Aripiprazole	Phenytoin	Imipramine	
Simvastatin	Metoprolol	Risperidone	Tramadol	Desipramine	
Rosuvastatin	Propafenone	Venlafaxine	Clomipramine	Haloperidol	
Pravastatin		Paroxetine	Fluvoxamine	Trimipramine	

**Table 2:** Selected pharmacogene–drug relationships included in the BioFRAM PGx framework. For each pharmacogene, the table presents the relevant predicted phenotype or functional category, the variants or alleles evaluated within BioFRAM PGx, the associated drug or drugs, and the corresponding CPIC and DPWG prescribing source available at the December 2022 evidence cut-off. All included pharmacogene–drug relationships met the ClinPGx Level 1A clinical annotation criterion at that cut-off. Colours indicate pharmacogene class: green, cytochrome P450 enzymes; yellow, drug transporters; and brown, VKORC1. Dashes (—) indicate that no corresponding prescribing source from that organization was identified for the relationship at the defined cut-off. TCA, tricyclic antidepressant; SSRI, selective serotonin reuptake inhibitor; CPIC, Clinical Pharmacogenetics Implementation Consortium; DPWG, Dutch Pharmacogenetics Working Group, (*) denote pharmacogene star-allele nomenclature.

Pharmacogene	Relevant Phenotype	Variants/Alleles Evaluated	Selected Drug	CPIC Prescribing Source	DPWG Prescribing Source
*CYP2D6*	Poor, intermediate, normal and ultrarapid metabolizer (activity-score based)	*1, *2, *3, *4, *5, *6, *9, *10, *17, *29, *41; copy number (*1xN, *2xN)	Flecainide	—	DPWG drug-specific recommendation
			Metoprolol	—	DPWG drug-specific recommendation
			Propafenone	—	DPWG drug-specific recommendation
			Amitriptyline	CPIC tricyclic-antidepressant guideline, 2016 update	DPWG TCA recommendation
			Clomipramine	CPIC tricyclic-antidepressant guideline, 2016 update	DPWG TCA recommendation
			Desipramine	CPIC tricyclic-antidepressant guideline, 2016 update	DPWG TCA recommendation
			Doxepin	CPIC tricyclic-antidepressant guideline, 2016 update	DPWG TCA recommendation
			Imipramine	CPIC tricyclic-antidepressant guideline, 2016 update	DPWG TCA recommendation
			Nortriptyline	CPIC tricyclic-antidepressant guideline, 2016 update	DPWG TCA recommendation
			Trimipramine	CPIC tricyclic-antidepressant guideline, 2016 update	DPWG TCA recommendation
			Aripiprazole	—	DPWG antipsychotic guidance/assessment
			Atomoxetine	CPIC atomoxetine guideline, 2019	DPWG atomoxetine recommendation
			Fluvoxamine	CPIC SSRI guideline, 2015	DPWG SSRI guideline
			Haloperidol	—	DPWG antipsychotic guidance/assessment
			Vortioxetine	—	DPWG drug-specific guidance/assessment
			Paroxetine	CPIC SSRI guideline, 2015	DPWG SSRI guideline
			Risperidone	—	DPWG antipsychotic guidance/assessment
			Tramadol	CPIC opioid guideline, 2020	DPWG opioid recommendation
			Venlafaxine	—	DPWG non-SSRI/non-TCA antidepressant recommendation
			Zuclopenthixol	—	DPWG antipsychotic guidance/assessment
*CYP2C19*	Poor, intermediate, normal, rapid and ultrarapid metabolizer	*1, *2, *3, *4, *17	Clopidogrel	CPIC clopidogrel guideline, 2022	DPWG clopidogrel recommendation
			Escitalopram	CPIC SSRI guideline, 2015	DPWG SSRI guideline
			Citalopram	CPIC SSRI guideline, 2015	DPWG SSRI guideline
			Sertraline	CPIC SSRI guideline, 2015	DPWG SSRI guideline
			Amitriptyline	CPIC tricyclic-antidepressant guideline, 2016 update	DPWG TCA recommendation
			Clomipramine	CPIC tricyclic-antidepressant guideline, 2016 update	DPWG TCA recommendation
			Doxepin	CPIC tricyclic-antidepressant guideline, 2016 update	DPWG TCA recommendation
			Imipramine	CPIC tricyclic-antidepressant guideline, 2016 update	DPWG TCA recommendation
			Trimipramine	CPIC tricyclic-antidepressant guideline, 2016 update	DPWG TCA recommendation
*CYP2C9*	Poor, intermediate and normal metabolizer (activity-score based)	*1, *2, *3	Warfarin	CPIC warfarin guideline, 2017	DPWG vitamin K antagonist recommendation
			Fluvastatin	CPIC statin guideline, 2022	—
			Phenytoin	CPIC phenytoin guideline, 2020	DPWG phenytoin recommendation
*CYP3A4*	Normal versus decreased CYP3A4 activity/function	*1, *22	Quetiapine	—	DPWG antipsychotic guidance/assessment
*SLCO1B1*	Normal, decreased or poor transporter function	*1, *5	Atorvastatin	CPIC statin guideline, 2022	DPWG SLCO1B1–statin guidance/assessment
			Fluvastatin	CPIC statin guideline, 2022	DPWG SLCO1B1–statin guidance/assessment
			Lovastatin	CPIC statin guideline, 2022	DPWG SLCO1B1–statin guidance/assessment
			Pitavastatin	CPIC statin guideline, 2022	DPWG SLCO1B1–statin guidance/assessment
			Pravastatin	CPIC statin guideline, 2022	DPWG SLCO1B1–statin guidance/assessment
			Rosuvastatin	CPIC statin guideline, 2022	DPWG SLCO1B1–statin guidance/assessment
			Simvastatin	CPIC statin guideline, 2022	DPWG SLCO1B1–statin guidance/assessment
*ABCG2*	Normal, decreased or poor transporter function	rs2231142	Rosuvastatin	CPIC statin guideline, 2022	—
*VKORC1*	*VKORC1* expression/vitamin K antagonist sensitivity category	rs9923231	Acenocoumarol	—	DPWG vitamin K antagonist recommendation
			Warfarin	CPIC warfarin guideline, 2017	DPWG vitamin K antagonist recommendation

**Table 3:** Summary of clinical, laboratory, and patient-reported data domains proposed for collection within the BioFRAM PGx framework.

Data Category	Variable Group
Sociodemographic and Lifestyle Characteristics	Sociodemographic data
	Lifestyle habits
	Psychiatric history
Clinical Characteristics	Anthropometrics and vital signs
	Health-related quality of life
Clinical Signs and Symptoms	Cardiovascular
	Neurological and neuropsychiatric
	Musculoskeletal
	Gastrointestinal
	Genitourinary and sexual function
	Sensory organs
	Skin and hepatic signs
Medical History and Treatments	Medical history
	Concomitant treatments
	Health care utilization
Laboratory Assessments	Haematology and coagulation
	Metabolic and renal function
	Liver function and proteins
	Lipid profile
	Electrolytes and tissue markers
	Inflammation and immunology

**Table 4:** Selected *CYP2D6* and *CYP2C9* alleles included in the BioFRAM PGx panel, together with their functional classification and corresponding activity value used for predicted-phenotype assignment. (*) denote pharmacogene star-allele nomenclature. “x” denotes multiplication in copy-number notation.

Gene	Alleles Included	Allele Function	Assigned Activity Value
*CYP2D6*	*3, *4, *5, *6	No function	0
	*9, *10, *41	Decreased function	0.25
	*17, *29		0.5
	*1, *2	Normal function	1
	*1xN, *2xN	Increased function	1xN
*CYP2C9*	*3	No function	0
	*2	Decreased function	0.5
	*1	Normal function	1

## Data Availability

The original contributions presented in this study are included in the article/Supplementary Material. Further inquiries can be directed to the corresponding author.

## Supplementary Materials

The following supporting information can be downloaded at: https://www.mdpi.com/article/10.3390/ph19081305/s1, Table S1: Genetic variants and structural alterations targeted within *CYP2D6*; Table S2: Genetic variants targeted within *CYP2C19*; Table S3: Genetic variants targeted within *CYP2C9*, *SLCO1B1*, and *CYP3A4*; Table S4: Genetic variants targeted within *VKORC1* and *ABCG2*; Table S5: Sociodemographic characteristics, lifestyle habits, psychiatric history, anthropometric measures, vital signs, and health-related quality-of-life variables collected at study enrolment; Table S6: Clinical signs, symptoms, cardiovascular history, and electrocardiographic variables assessed in the study; Table S7: Medical history, concomitant pharmacological treatments, and health care utilization variables collected in the study; Table S8: Laboratory measurements obtained from routine clinical laboratory records; Table S9: Mapping of pharmacogenetically relevant drugs and therapeutic groups to drug-specific clinical outcome domains and corresponding variables available within the BioFRAM PGx framework; the Membership of the BioFRAM PGx Consortium.

## Abbreviations

The following abbreviations are used in this manuscript:

PGx	Pharmacogenetics
gBMs	Pharmacogenetic biomarkers
ADRs	Adverse drug reactions
NHS	National Health System
EMA	European Medicines Agency
CPIC	Clinical Pharmacogenetics Implementation Consortium
DPWG	Dutch Pharmacogenetics Working Group
ATC	Anatomical Therapeutic Chemical
WMA	World Medical Association
eCRF	Electronic Case Report Form
REDCap	Research Electronic Data Capture platform
PM	Poor metabolizer
IM	Intermediate metabolizer
NM	Normal metabolizer
UM	Ultrarapid metabolizer
AS	Activity Score
