# Supplementary material for: BioFRAM PGx, an Implementation Proposal for Pharmacogenetics in the Spanish National Health System
Source: Pharmaceuticals (Basel). 2026 Aug 18;19(8):1305. doi: 10.3390/ph19081305 (PMC13516022; doi:10.3390/ph19081305)
Supplement: Supplementary file 1 [file pharmaceuticals-19-01305-s001.zip › pharmaceuticals-4429529-BioFRAM PGx Implementation Consortioum.pdf]

Extremadura: Francisco Arias-Aragón [1], Carmen Mata-Martín [1, 2], Alba Sánchez-Redondo [1], Pedro Dorado Hernández [1], María Estévez Paredes [2], Eva María Peñas Lledó [1], Beatriz Grillo Durán [1], Carla González De La Cruz [1], Ignacio Torres Solis [3], María Reyes González Fernández [4], Francisco Javier Lillo Bravo [5], José Luis Vicente Torrecilla [5], Félix Suárez González [6], Idilio González Martínez [7, 8], María Suárez Gómez [9], Carlos Barragán Gómez Coronado [5, 6, 10], Idian González Rodríguez [3] , Ana Bravo Pablo [11], Francisco Buitrago Ramírez [5], Mª Eugenia Fuentes Cañamero [12], José Manuel Ramiro Lozano [13], Carmen María González de la Portilla-Concha [14], Adrián Llerena [1, 2].
[1] Extremadura Biosanitary Research Institute (INUBE), 06080 Badajoz, Spain
[2] Pharmacogenetic and Personalised Medicine Unit, Extremadura Health Service, Badajoz University Hospital, Extremadura University, 06080 Badajoz, Spain
[3] Psychiatry Unit, Cáceres University Hospital, Extremadura Health Service, 10004 Cáceres, Spain.
[4] Cardiology Department, Badajoz University Hospital, Extremadura Health Service, 06080 Badajoz, Spain.
[5] La Paz Primary Care Health Centre, Extremadura Health Service, 06080 Badajoz, Spain.
[6] San Roque Primary Care Health Centre, Extremadura Health Service, 06080 Badajoz, Spain.
[7] Mérida Primary Care Health Centre, Extremadura Health Service, 06800 Mérida, Spain.
[8] Psychiatry Unit, Llerena Hospital, Extremadura Health Service, 06900 Llerena, Spain.
[9] Mérida (north) Primary Care Health Centre, Extremadura Health Service, 06800 Mérida, Spain.
[10] San Roque Primary Care Health Centre, Extremadura Health Service, 06080 Badajoz, Spain.
[11] Valdesalor-Aldea Moret Primary Care Health Centre, Extremadura Health Service, 10164 Cáceres, Spain.
[12] Cardiology Department, Hereditary Heart Disease Unit, Badajoz University Hospital, Extremadura Health Service, 06080 Badajoz, Spain.
[13] Internal Medicine Department, Lipid Unit, Badajoz University Hospital, Extremadura Health Service, 06080 Badajoz, Spain.
[14] Cardiology Department, Electrophysiology Unit, Badajoz University Hospital, Extremadura Health Service, 06080 Badajoz, Spain.

Madrid: Jesús Novalbos [1]; Miguel Ángel Seguido [1]; Paula Guerrero De La Riva [2]; Sara Moreda Meleiro [2]; Paloma Casado De Pedro [2]; Teresa Carmen Isasia Tortosa [2]; Tatiana Lozano Fernández [2]; María José Blázquez Martínez [2]; Julia Martínez Alfonso [2]; Rocío Arriaza Rubio [2]; Antonio Cabrera Majada [2]; Nuria Jiménez Olivas [2]; Eva De Santiago Cortés [2]; María Vega Manganiello [2]; Lidia De Prado Prieto [2]; Manuel De Frías Vargas [2]; Beatriz Cabrera Jurado [2]; Raquel González Román [2]; Susana Herrero Yusta [2]; Jorge Berzal Llorente [2]; Carmen Domínguez Encinas [2]; Natalia Martín Alonso [2]; Noelia Polo Fernández [3]; Jesús Gorosabel Rebolleda [3]; María Teresa Herranz Herrero [3]; Miriam Ordoñez Vicente [4]; Elena Pascual Martínez [4]; Elena Barrios Rueda [4]; Ana Isabel Muñoz García [4]; Gema Lineros Domínguez [4]; Carlos Jiménez Martínez [5]; Inés Sáenz Cardenal [5]; Lourdes Arias Egido [5]; Francisco Javier Cárdenas García [5]; Bárbara González Mancebo [5]; Ana Domínguez Rodríguez [5]; Alicia Fidalgo Rodríguez [5]; Paula Fernández Gorostieta [6]; M Carmen López Olmo [6]; M Rocío Sastre De La Fuente [7]; M Trinidad Sanz De La Fuente [7]; Fabiola Blanco Urzaiz [7]; Eugenia García Virosta [7]; Jorge Hernández Del Real [7]; Ana Carmen Cisneros Javierre [7]; Sarai Martin Álvarez [7]; Ana Ortega Soudant [7]; Sonia Vázquez Fuertes [7]; Beatriz Cava Sumner [7]; Nadia Kamal Moreno [7]; Lucia González Gómez [7]; María Concepción Cerrada Somolinos [8]; Cruz Calvete Modrego [8]; Mar Vizcaíno Blázquez [8]; Rosa Castro Esteban [8]; María José Cerezo Herrero [8]; Laura Olivo Alcaraz [8]; Beatriz García García [9]; Ana Alesón Andrés [9]; Markus Muizulis Alonso [9]; María Teresa Sarriá Sánchez [9]; María Luisa Revuelta Puig [9]; María Carmen Colorado Marín [9]; Isabel Gutiérrez Sánchez [10]; Teresa Mantilla

Morato [10]; Jorge Salamanca Viloria [11]; Pablo Díez Villanueva [11]; Clara Méndez Ponce [11]; Alejandra Gullón Ojesto [12]; María Del Mar Contreras Muruaga [12]; Patricia Ibáñez Sanz [12]; Emilia Roy Vallejo [12]; Noemí Gilabert González [12]; Ignacio De Los Santos Gil [12]; Andoni Casen Gil [12]; Isabel Belmonte Martín De Santa Olalla [12]; Lucio Jesus García-Fraile Fraile [12]; Azucena Bautista Hernández [12]; Ángela Gutiérrez Liarte [12]; María Aguilera García [12]; Carmen Sáez Béjar [12]; Ana Barrios Blandino [12]; Violeta Sampériz Rubio [12]; Itziar Leal Leturia [13]; Iosune Torio Palmero [13]; Fabio Mejías Fernández [1]; Raúl Parra Garcés [1]; Antía Gómez Fernández [1]; Cristina Ramos Del Moral [1]; Susana Almenara De Riquer [1]; Lucía Cañamero García [1]; Gina Paola Mejía Abril [1]; Feruza Muratova [1]; Karem Paola Rincón García [1]; Alejandra Luisa Vega Bonet [1]; Natali Giselle Lara González [1]; Edgardo Raúl Lovell Gaspari [1]; Luisa María Rojas Herrera [1]; Alejandro José Díaz Del Gobbo [1]; Marcos Gómez Navares [1]; Andrea Rodríguez López [1]; Carmen Rabadán Ungo [1]; Eva González Iglesias [1]; Paula Soria Chacartegui [1]; Francisco Abad-Santos [1].
[1] Clinical Pharmacology Department, Hospital Universitario de La Princesa, Pharmacology Department, Faculty of Medicine, Universidad Autónoma de Madrid, Instituto de Investigación Sanitaria la Princesa (IIS-Princesa), 28006 Madrid, Spain.
[2] Daroca Primary Care Health Centre, 28017 Madrid, Spain.
[3] Lagasca Primary Care Health Centre, 28006 Madrid, Spain.
[4] Londres Primary Care Health Centre, 28028 Madrid, Spain.
[5] Montesa Primary Care Health Centre, 28006 Madrid, Spain.
[6] Segre Primary Care Health Centre, 28002 Madrid, Spain.
[7] Santa Hortensia Primary Care Health Centre, 28002 Madrid, Spain.
[8] Castelló Primary Care Health Centre, 28009 Madrid, Spain.
[9] Baviera Primary Care Health Centre, 28028 Madrid, Spain.
[10] Prosperidad Primary Care Health Centre, 28028 Madrid, Spain.
[11] Cardiology Department, La Princesa University Hospital, 28006 Madrid, Spain.
[12] Internal Medicine Department, La Princesa University Hospital, 28006 Madrid, Spain.
[13] Psychiatry Department, La Princesa University Hospital, 28006 Madrid, Spain.

Cataluña: Mar Hernaez [1]; Eduard Vieta [2]; Rosa Catalán [2]; Antonio Benabarre [2]; María Luisa Imáz [2]; Eduard Parellada [2]; Miquel Bióque [2]; Emilio Ortega [3]; Rut Andrea [4]; Judit Julian Pena [5]; Vicent Llorca Bofí [6]; Mercè Brunet [7].
[1] Pharmacology and Toxicology Laboratory, Biochemistry and Molecular Genetics Department, Biomedical Diagnostic Center, Hospital Clinic of Barcelona, University of Barcelona, Fundació Clínic per a la Recerca Biomèdica (FCRB), 08036 Barcelona, Spain.
[2] Department of Psychiatry and Psychology, Hospital Clínic of Barcelona. University of Barcelona. Fundació de Recerca Clínic Barcelona-Institut d'Investigacions Biomèdiques August Pi i Sunyer (FCRB-IDIBAPS). Centro de investigación biomédica en red salud mental CIBERSAM, 08036 Barcelona, Spain
[3] Department of Endocrinology and Nutrition, Hospital Clínic of Barcelona. University of Barcelona. Fundació de Recerca Clínic Barcelona-Institut d'Investigacions Biomèdiques August Pi i Sunyer (FCRB-IDIBAPS). Centro de Investigación Biomédica en Red de la Fisiopatología de la Obesidad y Nutrición (CIBEROBN), 08036 Barcelona, Spain.
[4] Department of Cardiology, Hospital Clínic of Barcelona. University of Barcelona. Fundació de Recerca Clínic Barcelona-Institut d'Investigacions Biomèdiques August Pi i Sunyer (FCRB-IDIBAPS). Centro de Investigación Biomédica en Red de la Fisiopatología de la Obesidad y Nutrición (CIBEROBN), 08036 Barcelona, Spain.
[5] Department of Pharmacology and Toxicology, Hospital Clínic of Barcelona. University of Barcelona. Fundació de Recerca Clínic Barcelona-Institut d'Investigacions Biomèdiques August Pi i Sunyer (FCRB-IDIBAPS). Centro de Investigación Biomédica en Red de la Fisiopatología de la Obesidad y Nutrición (CIBEROBN), 08036 Barcelona, Spain.

[6] Barcelona Clínic Schizophrenia Unit (BCSU), Neuroscience Institute, Hospital Clínic of Barcelona; Institut D'Investigacions Biomèdiques August Pi i Sunyer (IDIBAPS); Centro de Investigación Biomédica en Red de Salud Mental, Instituto de Salud Carlos III (CIBERSAM); Department of Medicine, University of Barcelona, 08035 Barcelona, Spain.
[7] Pharmacology and Toxicology Laboratory, Biochemistry and Molecular Genetics Department, Biomedical Diagnostic Center, Hospital Clinic of Barcelona, University of Barcelona, FCRB-IDIBAPS, CIBERehd, 08036 Barcelona, Spain.

Castilla y León: David Heredero-Jung [1, 2]; Alejandro de la Sota-Pérez [1]; Belén García-Berrocal [1, 2]; Sandra Milagros Lorenzo-Hernández [2]; Elena Marcos-Vadillo [1, 2]; Pedro Luis Sánchez Fernández [3]; Eduardo Villacorta Arguelles [3]; Armando Oterino Manzanas [3]; Daniel Nieto Ibáñez [3]; Pedro Urbiola Sáez [3]; Giuseppe Barberís Barcia [3]; Juan Lorenzo Fraile [3]; María García de Pedro [3]; Jesús Pérez Sánchez-Toledo [4]; Carolina Concepción Lorenzo Romo [4]; Pablo Salas Aranda [4]; Manuel Ángel Franco Martín [5]; Paula Gutiérrez Baez [5]; Marcos González Iglesias [5]; Vicente Sáez Enguídanos [5]; Amaya Alonso Caldero [5]; María Isidoro-García [1, 2, 6].
[1] Institute of Biomedical Research of Salamanca (IBSAL), 37007 Salamanca, Spain.
[2] Department of Clinical Laboratory, Salamanca University Hospital (HUS), 37007 Salamanca, Spain.
[3] Department of Cardiology, University Hospital of Salamanca, 37007 Salamanca, Spain.
[4] Department of Psychiatry, University of Salamanca, 37007 Salamanca, Spain.
[5] Psychiatry and Mental Health Department, Healthcare Complex of Zamora, 49022 Zamora, Spain.
[6] Department of Medicine, University of Salamanca, 37007 Salamanca, Spain.

Galicia: Almudena Gil-Rodriguez [1, 2]; Alba Barral-Raña [1, 2]; Sheila Recarey-Rama [1, 3]; Ana Rodríguez-Viyuela [3, 4]; María Vidal-Millares [5, 6]; Ángela J. Torres [5, 6]; Ramón Ramos-Ríos [5]; Nuria Otero-Lago [5]; María José Durán-Maseda [5]; Verónica Prado-Robles [5]; Fernando Facal [5, 7]; Álvaro Fernández Monterroso [5]; Alberte Araúxo Vilar [5]; Alberto Ramos-Caneda [5]; María Elena Caruezo Rodríguez [5]; Manuel Arrojo [5, 6, 7]; Eduardo Echarri Arrieta [8]; Mario Páramo [5, 7]; Paloma Sempere-Serrano [8]; Diego Caicedo Valdés [9, 10]; Rita Calviño López-Villalta [9]; Carola Rubio Taboada [9]; Jorge Torre Eiriz [9]; María Brión [11, 12]; Rosa María Agra Bermejo [13, 14, 15]; Eva Ramos-Luis [11, 12]; María Álvarez-Barredo [12, 13, 14]; Marta Souto-Nieves [5]; Susana García-Luego Álvarez [5]; María Isabel Carballude García [5]; Ana María Mouta Otero [5, 6]; Iván Iglesias Carballo [5]; Cristina Antorrena Fernández [5]; Uxía Carles Magariños [5]; María José Fungueiriño Castro [5]; Olalla Maroñas [1, 2, 11].
[1] Pharmacogenomics and Drug Discovery Group (GenDeM), Health Research Institute of Santiago de Compostela (IDIS), Santiago de Compostela 15706, Spain.
[2] Genomics and Bioinformatics Group, Centre for Research in Molecular Medicine and Chronic Diseases (Ci-MUS), University of Santiago de Compostela, Santiago de Compostela 15782, Spain.
[3] Genomic Medicine Group, CIMUS, University of Santiago de Compostela, 15782 Santiago de Compostela, Spain.
[4] Genetics Group, Health Research Institute of Santiago de Compostela (IDIS), 15782 Santiago de Compostela, Spain.
[5] Department of Psychiatry, University Hospital of Santiago de Compostela, Galician Health Service (SERGAS), 15782 Santiago de Compostela, Spain.
[6] Department of Psychiatry, Radiology, Public Health, Nursing and Medicine, University of Santiago de Compostela, 15782 Santiago de Compostela, Spain.
[7] Psychiatric Genetics Group, Health Research Institute of Santiago de Compostela (IDIS), 15782 Santiago de Compostela, Spain.

[8] Department of Pharmacy, University Hospital of Santiago de Compostela, Galician Health Service (SERGAS), 15706 Santiago de Compostela, Spain.
[9] Department of Angiology and Vascular Surgery, University Hospital of Santiago de Compostela, Galician Health Service (SERGAS), 15706 Santiago de Compostela, Spain.
[10] Angiology and Vascular Surgery Group (VARGROS), Health Research Institute of Santiago de Compostela (IDIS), 15706 Santiago de Compostela, Spain.
[11] Galician Public Foundation of Genomic Medicine (FPGMX), Galician Health Service (SERGAS), Santiago de Compostela 15706, Spain.
[12] Cardiovascular Genetics Group, Health Research Institute of Santiago de Compostela (IDIS), 15706 Santiago de Compostela, Spain.
[13] Department of Cardiology, University Hospital of Santiago de Compostela, Galician Health Service (SERGAS), 15706 Santiago de Compostela, Spain.
[14] CIBER Cardiovascular, Instituto de Salud Carlos III, 28029 Madrid, Spain.
[15] Cardiology Research Group, Health Research Institute of Santiago de Compostela (IDIS), 15706 Santiago de Compostela, Spain.

Comunidad Valenciana: Gladys Guadalupe Olivera Pasquini [1]; Enrique G. Zucchet [1]; Luis Sendra Gisbert [1]; Gustavo Vila Castillo [2]; Ismael Ejarque Domenech [3, 4]; José Martinez Raga [5, 6]; Josep Andreu Pena Garijo [5]; José Vicente Cervera [7, 8]; Juan Silvestre Oltra Soler [8, 9]; Karl Knecht Armengod [5]; Luis Miguel Rojo Bofill [6, 10]; Pablo Navalon Rodriguez [6, 10]; Pilar Sierra San Miguel [6, 10]; Raquel Lopez Vilella [11]; Luis Almenar Bonet [6, 11, 12]; Victor Donoso Trenado [11]; Alicia Serrano Romero [13]; Vicent Bodi Peris [6, 12, 14, 15]; Victor Marcos Garcés [12, 14, 15]; Cesar Rios Navarro [12, 15]; Héctor Merenciano González [12, 14, 15]; Marina Berenguer Haym [6, 16, 17]; Patrice Gomes Marques [1, 16]; Mateo Campillo [5]; Amparo Lucia Aznar Basset [18]; Ana Blasco Romero [18]; Julia De La Fuente Arevalo [18]; Gloria Lopez Gomez [18]; Marta Llorens Garduño [18]; Manuela Vicente Cañizares [18]; Martín Larrea Solís [18]; María José Herrero[1].
[1] Department of Pharmacology, Universitat de València; Pharmacogenetics and Gene Therapy Unit, Instituto Investigación Sanitaria La Fe (IIS La Fe); 46026 Valencia, Spain.
[2] Gandia Primary Care Health Center (Corea), Gandía, Generalitat Valenciana Health Department (GVA), 46701 Gandia, Spain.
[3] Almácera Primary Care Health Center, Generalitat Valenciana Health Department (GVA), 46132 Almàssera Spain.
[4] Vithas Aguas Vivas Hospital, Carcaixent, 46740 Valencia, Spain.
[5] Department of Psychiatry and Mental Health, University Hospital Dr. Peset, Valencia, Spain.
[6] Department of Medicine, University of Valencia, 46017 Valencia, Spain.
[7] Department of Hematology and Hematology Research Group, Hospital Universitari i Politècnic La Fe, Health Research Institute La Fe (IIS La Fe), Valencia; Biomedical Research Networking Center in Cancer (CIBERONC), 46026 Valencia Spain.
[8] Genetics Unit, Hospital Universitari i Politècnic La Fe, 46010 Valencia, Spain.
[9] Department of Genetics, University of Valencia, 46010 Valencia, Spain.
[10] Department of Psychiatry, Mental Health Research Group, Hospital Universitari i Politècnic La Fe, Health Research Institute La Fe (IIS La Fe), 46026 Valencia, Spain.
[11] Department of Cardiology, Heart Failure and Transplant Unit, Hospital Universitari i Politècnic La Fe, 46010 Valencia, Spain.
[12] Biomedical Research Networking Center in Cardiovascular Diseases (CIBER-CV), Spain.
[13] Department of Cardiology, Hospital Universitari i Politècnic La Fe, 46010 Valencia, Spain.
[14] Department of Cardiology, Hospital Clínico Universitario de Valencia, 46010 Valencia, Spain.
[15] INCLIVA Health Research Institute, 46010 Valencia, Spain.
[16] Department of Hepatology, Hepatology and Liver Transplant Unit, Hospital Universitari i Politècnic La Fe, Health Research Institute La Fe (IIS La Fe), 46010 Valencia, Spain.

[17] Biomedical Research Networking Center in Liver and Digestive Diseases (CIBEREHD), Instituto de Salud Carlos III, 08036 Barcelona, Spain.
[18] Malilla Primary Care Health Center, Generalitat Valenciana (GVA), 46026 Valencia, Spain.

Andalucía: José Manuel Dodero Anillo [1]; Alicia Alba Máñez [1]; Marta Fernández-Pujol Marzo [1]; Laura Bello Bello [1]; Juan Bautista Raffo Nogueira [1]; Susana Herrera Caballero [2]; María del Mar Espinosa Nogales [3]; Consolación Montaña Tato [4]; Pilar Lozano Sanjuan [4]; Rosario Reguero Fernández [2]; Joaquina Lloreda Morillo [5]; Nazaret Marín Basallote [2]; Marina Bibiloni Martínez [4]; Rocío Martín Romero [3]; José Indalecio Ramírez Benítez [5]; María José Pedrosa Martínez [1].

[1] Clinical Pharmacology Unit, Hospital Universitario de Puerto Real, 11510 Puerto Real, Spain.
[2] Mental Health Inpatient Unit, Hospital Universitario de Puerto Real, 11510 Puerto Real, Spain.
[3] Community Mental Health Unit, Chiclana, Hospital Universitario de Puerto Real, 11510 Puerto Real, Spain.
[4] Community Mental Health Unit, Bahía, Hospital Universitario de Puerto Real, 11510 Puerto Real, Spain.
[5] Therapeutic Community for Mental Health, Hospital Universitario de Puerto Real, 11510 Puerto Real, Spain.
