# Supplementary material for: BioFRAM PGx, an Implementation Proposal for Pharmacogenetics in the Spanish National Health System
Source: Pharmaceuticals (Basel). 2026 Aug 18;19(8):1305. doi: 10.3390/ph19081305 (PMC13516022; doi:10.3390/ph19081305)
Supplement: Supplementary file 1 [file pharmaceuticals-19-01305-s001.zip › pharmaceuticals-4429529-supplementary.pdf]

pharmaceuticals

MDPI

Supplementary Material

Table S1. Genetic variants and structural alterations targeted within *CYP2D6*. Variant positions are reported according to the PharmVar ATG-start coordinate system using RefSeqGene NG_008376.4. For each targeted variant, the rsID, variant type, relevant star-allele context, molecular impact, and corresponding allele-function category are provided. The listed variants define the targeted *CYP2D6* variant coverage of the BioFRAM panel and include copy-number/structural variation relevant to *CYP2D6**5 and gene duplications or multiplications. Because individual variants may occur in more than one star allele, functional classification refers to the corresponding allele context rather than necessarily to the isolated variant. Complex *CYP2D6* structural configurations may not be fully resolved by targeted genotyping; these analytical limitations and the handling of unresolved results are described in the main text.

***CYP2D6* (NG_008376.4)**					
**PharmVar ATG-start position**	**rsID**	**Variant type**	**Star allele context**	**Molecular impact**	**Allele function**
**100C>T**	rs1065852	SNV (missense)	*4; *10	p.Pro34Ser (P34S)	*4: No function; *10: Decreased function
**1022C>T**	rs28371706	SNV (missense)	*17	p.Thr107Ile (T107I)	Decreased function
**1660G>A + 1662G>C**	rs61736512 + rs1058164	MNV / linked substitutions	*29	p.Val136Ile (V136I)	Decreased function
**1708delT**	rs5030655	Single-nucleotide deletion (frameshift)	*6	p.Trp152fs (W152fs)	No function
**1847G>A**	rs3892097	SNV (splice defect)	*4	Splice defect	No function
**2550delA**	rs35742686	Single-nucleotide deletion (frameshift)	*3	p.Arg259fs (R259fs)	No function
**2616delAAG**	rs5030656	In-frame 3-bp deletion	*9	p.Lys281del (K281del)	Decreased function
**2851C>T**	rs16947	SNV (missense)	*2; *17; *29; *41	p.Arg296Cys (R296C)	*2: Normal function; *17/*29/*41: Decreased function
**4181G>C**	rs1135840	SNV (missense)	*2; *4; *10; *17; *29; *41	p.Ser486Thr (S486T)	Context-dependent across listed alleles
**2989G>A**	rs28371725	SNV (splice defect)	*41	Splice defect	Decreased function
**3184G>A**	rs59421388	SNV (missense)	*29	p.Val338Met (V338M)	Decreased function

**Copy number / structural variation**	—	CNV / structural variant	*5 deletion; duplications/multiplications such as *1xN or *2xN	Full-gene deletion or duplication/multiplication	*5: No function/multiplications: functional effect depends on the duplicated allele

Table S2. Genetic variants targeted within *CYP2C19*. Variant positions are reported according to the PharmVar ATG-start coordinate system using RefSeqGene NG_008384.3. The table provides the rsID, variant type, relevant star-allele context, molecular impact, and allele-function category for each targeted variant. The listed variants define the targeted *CYP2C19* variant coverage of the Bio-FRAM panel. Because some variants are shared by star alleles with different functional classifications, allele function is reported according to the relevant haplotype context rather than attributed to the isolated variant alone.

CYP2C19 **(NG_008384.3)**					
**PharmVar ATG-start position**	**rsID**	**Variant type**	**Star allele context**	**Molecular impact**	**Allele function**
**-806C>T**	rs12248560	SNV (promoter/regulatory)	*17; some *4 suballeles	Increased transcription / expression-associated promoter variant	*17: Increased function; *4 No function
**1A>G**	rs28399504	SNV (start-loss / initiation-codon variant)	*4	p.Met1Val (M1V); altered initiation codon	No function
**12662A>G**	rs12769205	SNV (splice defect)	*2	Splice defect	No function
**17948G>A**	rs4986893	SNV (nonsense)	*3	p.Trp212Ter (W212X)	No function
**19154G>A**	rs4244285	SNV (splice defect)	*2	Splice defect	No function
**80161A>G**	rs3758581	SNV (missense)	*1; *2; *3; *4; *17	p.Ile331Val (I331V)	*1: Normal function; *2/*3/*4: No function; *17: Increased function

Table S3. Genetic variants targeted within *CYP2C9*, *SLCO1B1*, and *CYP3A4*. Variant positions are reported according to the PharmVar ATG-start coordinate system using the indicated RefSeqGene sequences (*CYP2C9*, NG_008385.2; *SLCO1B1*, NG_011745.1; *CYP3A4*, NG_008421.1). For each variant, the rsID, variant type, relevant star-allele context, molecular impact, and allele-function category are shown. The variants listed constitute the targeted variant coverage of these genes within the BioFRAM panel; the table does not imply exhaustive coverage of all currently defined alleles for each pharmacogene.

Gene (Ref sequence)	PharmVar ATG-start position	rsID	Variant type	Star allele context	Molecular impact	Allele function
CYP2C9 (NG_008385.2)	3608C>T	rs1799853	SNV (missense)	*2	p.Arg144Cys (R144C)	Decreased function
	42614A>C	rs1057910	SNV (missense)	*3	p.Ile359Leu (I359L)	No function
SLCO1B1 (NG_011745.1)	37041T>C	rs4149056	SNV (missense)	*5; also present in *15 and other haplotypes	p.Val174Ala (V174A)	Decreased function
CYP3A4 (NG_008421.1)	15389C>T	rs35599367	SNV (intronic/regulatory)	*22	Reduced CYP3A4 expression/activity	Decreased function

Table S4. Genetic variants targeted within *VKORC1* and *ABCG2*. Variants are identified using standardized variant nomenclature and rsIDs, together with the indicated RefSeqGene sequences (*VKORC1*, NG_011564.1; *ABCG2*, NG_032067.2). The table summarizes variant type, molecular impact, and functional effect for each targeted variant. The listed variants constitute the targeted *VKORC1* and *ABCG2* variant coverage of the BioFRAM panel. Unlike the pharmacogenes presented in Tables S1–S3, these variants are not represented here using PharmVar ATG-start/star-allele nomenclature.

Gene (Ref sequence)	Variant nomenclature	rsID	Variant type	Molecular impact	Functional effect
VKORC1 (NG_011564.1)	c.-1639G>A	rs9923231	SNV (promoter/regulatory)	Reduced VKORC1 expression	Reduced expression
ABCG2 (NG_032067.2)	c.421C>A	rs2231142	SNV (missense)	p.Gln141Lys (Q141K)	Decreased transporter function

Table S5. Sociodemographic characteristics, lifestyle habits, psychiatric history, anthropometric measures, vital signs, and health-related quality-of-life variables collected at study enrolment. Variables are operationalized according to the proposed study case report form (CRD). Categorical variables are coded using predefined CRD categories unless otherwise specified. Health-related quality of life is assessed using the EQ-5D-5L instrument; its five dimensions (mobility, self-care, usual activities, pain/discomfort, and anxiety/depression) are recorded using the standard five-level response system (1–5), and overall self-rated health is recorded using the EQ visual analogue scale (EQ VAS; 0–100).

Domain	Subdomain	Variable	Operationalization	Unit / coding	Collection time point	Data source
Sociodemographic Data	Demographics	Date of birth	Participant date of birth as recorded in the clinical record.	Date (DD/MM/YYYY)	At enrolment	Electronic medical record / clinical interview
	Demographics	Sex	Sex recorded for the participant in the clinical record.	Categorical; predefined CRD categories	At enrolment	Electronic medical record / clinical interview
	Geographic origin	Country / province / city of birth	Place of birth recorded at the available geographic level.	Free text / specify	At enrolment	Clinical interview / electronic medical record
	Population background	Ethnic group	Participant ethnic or population group as collected in the study record.	Categorical; predefined CRD categories	At enrolment	Clinical interview / patient report
Lifestyle Habits	Caffeine	Number of caffeine-containing drinks per day	Average number of caffeine-containing drinks consumed per day.	Number/day	At enrolment	Clinical interview / patient report
	Smoking	Current smoker	Current tobacco smoking status at the time of assessment.	Yes/No	At enrolment	Clinical interview / patient report
	Smoking	Number of cigarettes per day	Average number of cigarettes smoked per day among current smokers.	Cigarettes/day	At enrolment	Clinical interview / patient report
	Smoking	Number of years of smoking	Total duration of tobacco smoking exposure.	Years	At enrolment	Clinical interview / patient report
	Alcohol	Number of alcohol intakes per week	Average number of alcoholic-drink intakes reported per week.	Intakes/week	At enrolment	Clinical interview / patient report
	Substance use	Illicit drug use	Current or relevant illicit drug use, with substance specified when applicable.	Yes/No; specify substance if Yes	At enrolment	Clinical interview / patient report
	Physical activity	Physical activity level	Participant physical activity level according to the categories used in the study record.	Categorical; predefined CRD levels	At enrolment	Clinical interview / patient report
	Other	Other relevant lifestyle habits	Other lifestyle factors considered clinically relevant and not captured in predefined fields.	Free text / specify	At enrolment	Clinical interview / study record
Psychiatric History	Suicidality	History of suicide attempts	Documented or self-reported history of one or more suicide attempts.	Yes/No	At enrolment (history prior to inclusion)	Electronic medical record / clinical interview
Psychiatric History	Suicidality	Number of suicide attempts	Total number of documented or self-reported suicide attempts before assessment.	Count	At enrolment (history prior to inclusion)	Electronic medical record / clinical interview
Anthropometrics	Body measurements	Weight	Body weight measured or recorded closest to the study assessment.	kg	At enrolment / closest clinically available measurement	Clinical measurement / electronic medical record
	Body measurements	Height	Participant height measured or recorded in the clinical record.	cm	At enrolment / baseline record	Clinical measurement / electronic medical record

	Body measurements	Body Mass Index (BMI)	Body mass index calculated from weight and height.	kg/m^2	At enrolment / derived from baseline measurements	Calculated from weight and height / electronic medical record
Vital Signs	Respiratory	Oxygen saturation	Peripheral oxygen saturation measured at clinical assessment.	%	At enrolment / when clinically measured	Clinical measurement / electronic medical record
	Blood pressure	Systolic blood pressure	Systolic arterial blood pressure measured during clinical assessment.	mmHg	At enrolment / when clinically measured	Clinical measurement / electronic medical record
	Blood pressure	Diastolic blood pressure	Diastolic arterial blood pressure measured during clinical assessment.	mmHg	At enrolment / when clinically measured	Clinical measurement / electronic medical record
	Heart rate	Heart rate	Heart rate measured during clinical assessment.	beats/min	At enrolment / when clinically measured	Clinical measurement / electronic medical record
Health-Related Quality of Life	EQ-5D-5L	Mobility	Mobility dimension of the EQ-5D-5L questionnaire	Ordinal; EQ-5D-5L levels 1–5	At enrolment	Clinical interview / patient report
	EQ-5D-5L	Self-care	Self-care dimension of the EQ-5D-5L questionnaire	Ordinal; EQ-5D-5L levels 1–5	At enrolment	Clinical interview / patient report
	EQ-5D-5L	Usual activities	Usual activities dimension of the EQ-5D-5L questionnaire	Ordinal; EQ-5D-5L levels 1–5	At enrolment	Clinical interview / patient report
	EQ-5D-5L	Pain / Discomfort	Pain/discomfort dimension of the EQ-5D-5L questionnaire	Ordinal; EQ-5D-5L levels 1–5	At enrolment	Clinical interview / patient report
	EQ-5D-5L	Anxiety / Depression	Anxiety/depression dimension of the EQ-5D-5L questionnaire	Ordinal; EQ-5D-5L levels 1–5	At enrolment	Clinical interview / patient report
	EQ-5D-5L	Self-declared health status	Participant's self-rated overall health recorded using the EQ visual analogue scale	Continuous score; 0–100	At enrolment	Clinical interview / patient report

Table S6. Clinical signs, symptoms, cardiovascular history, and electrocardiographic variables assessed in the study. Unless otherwise specified, signs and symptoms are recorded as binary variables (Yes/No) based on patient report at the study interview. Previous coronary ischemic disease/events and venous thromboembolic events refer to medical history prior to enrolment and are obtained from the electronic medical record and clinical interview. Electrocardiographic information is obtained from clinically available assessments, with the corrected QT interval (QTc) recorded in milliseconds.

Domain	Subdomain	Variable	Operationalization	Unit / coding	Collection time point	Data source
Cardiac Assessment	Electrocardiography	Electrocardiogram (ECG)	Electrocardiographic assessment available or performed as part of the clinical evaluation.	Binary; Yes/No	At the study interview / clinically available assessment	Clinical assessment / medical record
	Electrocardiography	QTc interval	Corrected QT interval obtained from the electrocardiogram.	ms	At the study interview / clinically available assessment	Electrocardiogram / medical record
Cardiovascular	Cardiovascular symptoms	Palpitations	Patient-reported presence of palpitations at the time of the study interview.	Binary; Yes/No	At the study interview	Clinical interview / patient report
	Cardiovascular symptoms	Chest pain	Patient-reported presence of chest pain at the time of the study interview.	Binary; Yes/No	At the study interview	Clinical interview / patient report
	Cardiovascular symptoms	Coronary ischemia	History of previous coronary ischemic disease/event before study enrolment.	Binary; Yes/No	At the study interview (medical history prior to enrolment)	Electronic medical record / clinical interview
	Cardiovascular symptoms	Venous thromboembolism	History of previous venous thromboembolic event before study enrolment.	Binary; Yes/No	At the study interview (medical history prior to enrolment)	Electronic medical record / clinical interview
	Cardiovascular symptoms	Peripheral coldness	Patient-reported presence of peripheral coldness at the time of the study interview.	Binary; Yes/No	At the study interview	Patient report
Neurological and Neuropsychiatric Assessment	Cognitive / Sleep	Difficulty concentrating	Patient-reported difficulty concentrating at the time of the study interview.	Binary; Yes/No	At the study interview	Clinical interview / patient report
	Cognitive / Sleep	Sleep quality	Patient-reported alteration or poor sleep quality at the time of the study interview.	Binary; Yes/No	At the study interview	Clinical interview / patient report
	Neurological symptoms	Dizziness	Patient-reported presence of dizziness at the time of the study interview.	Binary; Yes/No	At the study interview	Clinical interview / patient report
	Neurological symptoms	Tremor / Parkinsonism	Patient-reported presence of tremor or parkinsonism-related symptoms at the time of the study interview.	Binary; Yes/No	At the study interview	Clinical interview / patient report
	Neurological symptoms	Rigidity / Hypertonia	Patient-reported presence of rigidity or hypertonia at the time of the study interview.	Binary; Yes/No	At the study interview	Clinical interview / patient report
	Neurological symptoms	Gait disorder	Patient-reported presence of gait disturbance at the time of the study interview.	Binary; Yes/No	At the study interview	Clinical interview / patient report
Musculoskeletal Symptoms	Muscle-related symptoms	Myalgias	Patient-reported presence of muscle pain at the time of the study interview.	Binary; Yes/No	At the study interview	Clinical interview / patient report
	Muscle-related symptoms	Muscle weakness / fatigue	Patient-reported presence of muscle weakness or muscle-related fatigue at the time of the study interview.	Binary; Yes/No	At the study interview	Clinical interview / patient report

	Muscle-related symptoms	Muscle cramps	Patient-reported presence of muscle cramps at the time of the study interview.	Binary; Yes/No	At the study interview	Clinical interview / patient report
Functional Status and General Symptoms	General symptoms	Asthenia / Fatigue	Patient-reported presence of asthenia or fatigue at the time of the study interview.	Binary; Yes/No	At the study interview	Clinical interview / patient report
	General symptoms	Appetite alteration	Patient-reported alteration in appetite at the time of the study interview.	Binary; Yes/No	At the study interview	Clinical interview / patient report
Gastrointestinal Symptoms	Upper gastrointestinal	Dyspepsia	Patient-reported presence of dyspepsia at the time of the study interview.	Binary; Yes/No	At the study interview	Clinical interview / patient report
	Upper gastrointestinal	Epigastric pain	Patient-reported presence of epigastric pain at the time of the study interview.	Binary; Yes/No	At the study interview	Clinical interview / patient report
	Motility	Constipation	Patient-reported presence of constipation at the time of the study interview.	Binary; Yes/No	At the study interview	Clinical interview / patient report
	Motility	Diarrhoea	Patient-reported presence of diarrhoea at the time of the study interview.	Binary; Yes/No	At the study interview	Clinical interview / patient report
	Tolerance	Nausea / Vomiting	Patient-reported presence of nausea or vomiting at the time of the study interview.	Binary; Yes/No	At the study interview	Clinical interview / patient report
Genitourinary and Sexual Function	Genitourinary	Urinary retention	Patient-reported presence of urinary retention at the time of the study interview.	Binary; Yes/No	At the study interview	Clinical interview / patient report
	Sexual function	Sexual dysfunction	Patient-reported presence of sexual dysfunction at the time of the study interview.	Binary; Yes/No	At the study interview	Clinical interview / patient report
Sensory Organs	Auditory	Hearing loss	Patient-reported presence of hearing loss at the time of the study interview.	Binary; Yes/No	At the study interview	Clinical interview / patient report
	Ophthalmologic	Eye examination	Patient-reported presence of an eye-related abnormality or relevant finding as captured in the CRD.	Binary; Yes/No	At the study interview	Clinical interview / patient report
	Ophthalmologic	Mydriasis	Patient-reported presence of mydriasis at the time of the study interview.	Binary; Yes/No	At the study interview	Clinical interview / patient report
	Ophthalmologic	Nystagmus	Patient-reported presence of nystagmus at the time of the study interview.	Binary; Yes/No	At the study interview	Clinical interview / patient report
Skin, Mucosae and Hepatic Signs	Skin and mucosae	Dryness	Patient-reported presence of skin or mucosal dryness at the time of the study interview.	Binary; Yes/No	At the study interview	Clinical interview / patient report
	Skin and mucosae	Dry mouth	Patient-reported presence of dry mouth at the time of the study interview.	Binary; Yes/No	At the study interview	Clinical interview / patient report
	Skin and mucosae	Blisters / Wheals	Patient-reported presence of blisters or wheals at the time of the study interview.	Binary; Yes/No	At the study interview	Clinical interview / patient report
	Skin and mucosae	Skin necrosis	Patient-reported presence or history of skin necrosis as captured at the study interview.	Binary; Yes/No	At the study interview	Clinical interview / patient report
	Skin and mucosae	Other skin findings	Patient-reported presence of other relevant skin findings, including urticaria or bleeding, as captured in the CRD.	Binary; Yes/No; specify finding if applicable	At the study interview	Clinical interview / patient report
	Hepatic signs	Jaundice	Patient-reported presence of jaundice at the time of the study interview.	Binary; Yes/No	At the study interview	Clinical interview / patient report

Table S7. Medical history, concomitant pharmacological treatments, and health care utilization variables collected in the study. Diagnoses and treatment-related variables are obtained from the electronic medical record and coded according to the study CRD, including medication identification, ATC classification, route of administration, dose, dosing frequency, and treatment dates where available. Health care utilization variables refer to the predefined retrospective assessment window established in the study CRD and include primary care and emergency department visits, specialist care, and hospital admissions.

Domain	Subdomain	Variable	Definition / operationalization	Unit / coding	Collection time point	Data source
Medical History	Medical diagnoses	Number of medical diagnoses	Total number of relevant medical diagnoses recorded for the participant.	Count	At enrolment (medical history prior to inclusion)	Electronic medical record
	Diagnosis details	Diagnosis description	Clinical diagnosis recorded for the participant.	Free text	At enrolment (medical history prior to inclusion)	Electronic medical record
	Diagnosis details	Diagnosis start date	Date of onset or diagnosis, when available.	Date	At enrolment (retrospective medical history)	Electronic medical record
	Diagnosis details	Associated treatment	Treatment associated with the recorded diagnosis, when applicable.	Free text	At enrolment	Electronic medical record
Treatments	Medication identification	Medication name	Name of current or previous concomitant medication recorded in the study.	Text / coded medication name	At enrolment and according to treatment history recorded in the CRD	Electronic medical record
	Medication identification	ATC group	Anatomical Therapeutic Chemical classification associated with the recorded medication.	ATC code/category	At enrolment and according to treatment history recorded in the CRD	Electronic medical record
	Administration details	Route of administration	Route by which the medication is administered.	Categorical; predefined CRD categories	At enrolment and according to treatment history recorded in the CRD	Electronic medical record
	Administration details	Dose	Recorded dose of the medication per administration or according to the CRD dosing field.	Numeric + dose unit	At enrolment and according to treatment history recorded in the CRD	Electronic medical record
	Administration details	Dosing frequency	Frequency with which the medication is administered.	Administrations per day	At enrolment and according to treatment history recorded in the CRD	Electronic medical record
	Administration details	Treatment start date	Date on which treatment with the recorded medication started, when available.	Date	At enrolment / retrospective treatment history	Electronic medical record
	Administration details	Treatment end date	Date on which treatment ended, when applicable.	Date / Ongoing	At enrolment / retrospective treatment history	Electronic medical record
Health Care Utilization	Primary care visits	Number of primary care nursing/physician visits	Number of primary care nursing or physician visits during the predefined assessment window.	Count	Retrospective predefined assessment window	Electronic medical record
	Emergency care	Number of emergency department visits	Number of emergency department visits during the predefined assessment window.	Count	Retrospective predefined assessment window	Electronic medical record
	Specialist care	Medical specialties visited	Specialist medical services attended during the predefined assessment window.	Predefined CRD categories / multiple response	Retrospective predefined assessment window	Electronic medical record
	Hospitalizations	Number of hospitalizations	Number of hospital admissions during the predefined assessment window.	Count	Retrospective predefined assessment window	Electronic medical record

	Hospitalizations	Length of stay	Duration of hospitalization for each recorded admission.	Days	Retrospective predefined assessment window	Electronic medical record
	Hospitalizations	Reason for admission	Main clinical reason for each recorded hospital admission.	Free text	Retrospective predefined assessment window	Electronic medical record

Table S8. Laboratory measurements obtained from routine clinical laboratory records for the assessment of haematological, glycaemic, renal, hepatic, lipid, electrolyte, muscle/tissue, inflammatory, and immunological parameters. For each variable, the most recent available measurement within the 6 months prior to study enrolment is considered. Because BioFRAM is a multicentre framework and laboratory reporting practices may differ between participating centres, laboratory-specific units or coding are retained where applicable; fixed units are reported for variables with a standardized or consistently defined measurement scale.

Domain	Subdomain	Variable	Definition / operationalization	Unit / coding	Collection time point	Data source
Haematology	Red blood cells	Red blood cell count (RBC)	Peripheral blood red blood cell count.	Laboratory-specific unit	Most recent available measurement within the 6 months prior to study enrolment	Laboratory report / electronic medical record
	Red blood cells	Haemoglobin	Blood haemoglobin concentration.	Laboratory-specific unit	Most recent available measurement within the 6 months prior to study enrolment	Laboratory report / electronic medical record
	Red blood cells	Haematocrit	Proportion of blood volume occupied by red blood cells.	% or laboratory-specific equivalent	Most recent available measurement within the 6 months prior to study enrolment	Laboratory report / electronic medical record
	Red blood cells	Mean corpuscular volume (MCV)	Mean volume of individual red blood cells.	fL	Most recent available measurement within the 6 months prior to study enrolment	Laboratory report / electronic medical record
	White blood cells	White blood cell count (WBC)	Peripheral blood total leukocyte count.	Laboratory-specific unit	Most recent available measurement within the 6 months prior to study enrolment	Laboratory report / electronic medical record
	White blood cells	Neutrophils (%)	Relative proportion of neutrophils among circulating leukocytes.	%	Most recent available measurement within the 6 months prior to study enrolment	Laboratory report / electronic medical record
	White blood cells	Neutrophils (absolute count)	Absolute neutrophil count in peripheral blood.	Laboratory-specific unit	Most recent available measurement within the 6 months prior to study enrolment	Laboratory report / electronic medical record
	White blood cells	Lymphocytes (%)	Relative proportion of lymphocytes among circulating leukocytes.	%	Most recent available measurement within the 6 months prior to study enrolment	Laboratory report / electronic medical record
	White blood cells	Lymphocytes (absolute count)	Absolute lymphocyte count in peripheral blood.	Laboratory-specific unit	Most recent available measurement within the 6 months prior to study enrolment	Laboratory report / electronic medical record

	White blood cells	Eosinophils (%)	Relative proportion of eosinophils among circulating leukocytes.	%	Most recent available measurement within the 6 months prior to study enrolment	Laboratory report / electronic medical record
	Platelets	Platelet count	Peripheral blood platelet count.	Laboratory-specific unit	Most recent available measurement within the 6 months prior to study enrolment	Laboratory report / electronic medical record
	Coagulation	International Normalized Ratio (INR)	Standardized ratio of prothrombin time used to assess coagulation.	Dimensionless ratio	Most recent available measurement within the 6 months prior to study enrolment	Laboratory report / electronic medical record
Glycaemic Control	Glucose metabolism	Fasting plasma glucose	Plasma glucose concentration measured under fasting conditions.	Laboratory-specific unit	Most recent available measurement within the 6 months prior to study enrolment	Laboratory report / electronic medical record
	Glucose metabolism	Glycosylated haemoglobin (HbA1c)	Glycated haemoglobin reflecting average glycaemia over the preceding months.	Laboratory-specific unit	Most recent available measurement within the 6 months prior to study enrolment	Laboratory report / electronic medical record
Renal Function	Renal markers	Urea	Serum/plasma urea concentration.	Laboratory-specific unit	Most recent available measurement within the 6 months prior to study enrolment	Laboratory report / electronic medical record
	Renal markers	Creatinine	Serum/plasma creatinine concentration.	Laboratory-specific unit	Most recent available measurement within the 6 months prior to study enrolment	Laboratory report / electronic medical record
	Renal markers	Estimated glomerular filtration rate (eGFR)	Estimated glomerular filtration rate reported by the clinical laboratory.	mL/min/1.73 m^2	Most recent available measurement within the 6 months prior to study enrolment	Laboratory report / electronic medical record
	Urinalysis	Urine function	Urinalysis or urinary-function result as recorded in the study data.	Categorical	Most recent available measurement within the 6 months prior to study enrolment	Laboratory report / electronic medical record
	Urinalysis	Other urine abnormalities	Other clinically relevant urinary laboratory abnormalities captured in the CRD.	Free text	Most recent available measurement within the 6 months prior to study enrolment	Laboratory report / electronic medical record
Liver Function and Proteins	Liver enzymes	AST (GOT)	Aspartate aminotransferase activity.	Laboratory-specific unit	Most recent available measurement within the 6 months prior to study enrolment	Laboratory report / electronic medical record
	Liver enzymes	ALT (GPT)	Alanine aminotransferase activity.	Laboratory-specific unit	Most recent available measurement within the 6 months prior to study enrolment	Laboratory report / electronic medical record
	Liver enzymes	Gamma-glutamyl transferase (GGT)	Gamma-glutamyl transferase activity.	Laboratory-specific unit	Most recent available measurement within the 6 months prior to study enrolment	Laboratory report / electronic medical record

	Liver enzymes	Alkaline phosphatase (ALP)	Alkaline phosphatase activity.	Laboratory-specific unit	Most recent available measurement within the 6 months prior to study enrolment	Laboratory report / electronic medical record
	Liver function	Total bilirubin	Total serum/plasma bilirubin concentration.	Laboratory-specific unit	Most recent available measurement within the 6 months prior to study enrolment	Laboratory report / electronic medical record
	Proteins	Albumin	Serum/plasma albumin concentration.	Laboratory-specific unit	Most recent available measurement within the 6 months prior to study enrolment	Laboratory report / electronic medical record
Lipids and Cardiovascular Risk	Lipid profile	Triglycerides	Serum/plasma triglyceride concentration.	Laboratory-specific unit	Most recent available measurement within the 6 months prior to study enrolment	Laboratory report / electronic medical record
	Lipid profile	Total cholesterol	Serum/plasma total cholesterol concentration.	Laboratory-specific unit	Most recent available measurement within the 6 months prior to study enrolment	Laboratory report / electronic medical record
	Lipid profile	HDL cholesterol	High-density lipoprotein cholesterol concentration.	Laboratory-specific unit	Most recent available measurement within the 6 months prior to study enrolment	Laboratory report / electronic medical record
	Lipid profile	LDL cholesterol	Low-density lipoprotein cholesterol concentration.	Laboratory-specific unit	Most recent available measurement within the 6 months prior to study enrolment	Laboratory report / electronic medical record
Electrolytes and Other Biochemistry	Electrolytes	Sodium (Na^+)	Serum/plasma sodium concentration.	mmol/L	Most recent available measurement within the 6 months prior to study enrolment	Laboratory report / electronic medical record
	Electrolytes	Potassium (K^+)	Serum/plasma potassium concentration.	mmol/L	Most recent available measurement within the 6 months prior to study enrolment	Laboratory report / electronic medical record
	Muscle / tissue markers	Lactate dehydrogenase (LDH)	Serum/plasma lactate dehydrogenase activity.	Laboratory-specific unit	Most recent available measurement within the 6 months prior to study enrolment	Laboratory report / electronic medical record
	Muscle / tissue markers	Creatine kinase (CK)	Serum/plasma creatine kinase activity.	Laboratory-specific unit	Most recent available measurement within the 6 months prior to study enrolment	Laboratory report / electronic medical record
	Other metabolites	Uric acid	Serum/plasma uric acid concentration.	Laboratory-specific unit	Most recent available measurement within the 6 months prior to study enrolment	Laboratory report / electronic medical record
Inflammation and Immunology	Inflammation markers	C-reactive protein (CRP)	Serum/plasma C-reactive protein concentration.	Laboratory-specific unit	Most recent available measurement within the 6 months prior to study enrolment	Laboratory report / electronic medical record

Autoimmunity	Rheumatoid factor	Rheumatoid factor measurement as reported by the clinical laboratory.	Laboratory-specific coding	Most recent available measurement within the 6 months prior to study enrolment	Laboratory report / electronic medical record

Table S9. Mapping of pharmacogenetically relevant drugs and therapeutic groups to drug-specific clinical outcome domains and corresponding variables available within the BioFRAM framework. Variables and their operational definitions, coding, collection time points, and data sources are detailed in Tables S5–S8. Treatment discontinuation or switching is derived from medication records and is considered descriptive because the reason for treatment modification is not systematically recorded. Outcomes not directly captured by the CRD, including seizure control and a specific quantitative measure of analgesic response, are not inferred from treatment changes alone. UKU-derived items are used to characterize psychotropic treatment tolerability rather than therapeutic efficacy.

Drug / therapeutic group	Pharmacogene(s)	Outcome domain	Variables available in BioFRAM	Data source
Vitamin K antagonists (acenocoumarol, warfarin)	*CYP2C9; VKORC1*	Anticoagulation control and safety	INR; thromboembolic/bleeding-related events/ADRs; treatment start/end dates; treatment switching/discontinuation patterns	Laboratory report; electronic medical record; clinical interview; pharmacovigilance assessment
Clopidogrel	*CYP2C19*	Cardiovascular history and safety indicators	History of coronary ischemia; cardiovascular diagnoses/events when documented in the medical record; treatment switching/discontinuation patterns; ADRs	Electronic medical record; clinical interview; pharmacovigilance assessment
Statins	*SLCO1B1*; *ABCG2*; *CYP2C9* where applicable	Lipid response and musculoskeletal tolerability	LDL cholesterol; total cholesterol; triglycerides; CK; myalgias; muscle weakness/fatigue; muscle cramps; treatment switching/discontinuation patterns; ADRs	Laboratory report; electronic medical record; clinical interview/patient report; pharmacovigilance assessment
Antidepressants	*CYP2D6*; *CYP2C19*	Treatment tolerability and patient-reported health status	UKU-derived adverse-effect items; EQ-5D-5L dimensions and EQ VAS; treatment switching/discontinuation patterns; ADRs	Clinical interview/patient report; electronic medical record; pharmacovigilance assessment
Antipsychotics	*CYP2D6*; *CYP3A4*	Treatment tolerability and adverse effects	UKU-derived adverse-effect items; tremor/Parkinsonism; rigidity/hypertonia; gait disorder; weight; BMI; treatment switching/discontinuation patterns; ADRs	Clinical interview/patient report; clinical measurements; electronic medical record; pharmacovigilance assessment
Atomoxetine	*CYP2D6*	Cardiovascular and general tolerability indicators	Heart rate; systolic and diastolic blood pressure; appetite alteration; sleep quality; weight; BMI; treatment switching/discontinuation patterns; ADRs	Clinical interview/patient report; clinical measurements; electronic medical record; pharmacovigilance assessment
Tramadol	*CYP2D6*	Pain-related status and treatment pattern	EQ-5D-5L Pain/Discomfort; treatment switching/discontinuation patterns; ADRs	Clinical interview/patient report; electronic medical record; pharmacovigilance assessment
Phenytoin	*CYP2C9*	Neurological tolerability and treatment pattern	Dizziness; tremor/Parkinsonism; gait disorder; nystagmus; treatment switching/discontinuation patterns; ADRs	Clinical interview/patient report; electronic medical record; pharmacovigilance assessment
CYP2D6-related cardiovascular drugs (metoprolol, flecainide, propafenone)	*CYP2D6*	Cardiovascular response and safety indicators	Heart rate; systolic and diastolic blood pressure; ECG; QTc; palpitations; chest pain; treatment switching/discontinuation patterns; ADRs	Clinical assessment; ECG; electronic medical record; clinical interview/patient report; pharmacovigilance assessment
